# Maternal, perinatal, neonatal, and postpartum outcomes following Severe Acute Respiratory Syndrome - Coronavirus-2 infection in pregnancy: a World Health Organization multi-country prospective cohort study

**DOI:** 10.1186/s44263-026-00292-9

**Published:** 2026-08-10

**Authors:** WHO COVID-19 & Pregnancy Cohort Study Group, Edna Kara, Sami L. Gottlieb, Nathalie J. Broutet, Anna Thorson, Marie Delnord, Ibukun-Oluwa Omolade Abejirinde, Ronaldo Silva, Soe Soe Thwin, Daniel Giordano, Ndema Abu Habib, Edgardo Abalos, José G. Cecatti, Maria L. Costa, Renato T. Souza, Seni Kouanda, Désiré Lucien Dahourou, Henri Gautier Ouedraogo, Sergio Munoz, Gerardo Espinoza, Araceli Saavedra, Kwasi Torpey, Chris Guure, Ernest Maya, Emefa Modey, Marleen Temmerman, Rodney D. Adam, Ingrid Gichere, Sura Mandeep, Sarah Saleem, Najia Ghanchi, Saleem Jessani, Erlidia F. Llamas-Clark, Emmanuel S. Baja, Mayan U. Lumandas, Alejandro Orrico-Sánchez, Antonio Carmona, Henda Triki, Mariem Gdoura

**Affiliations:** 1 UNDP/UNFPA/UNICEF/WHO/World Bank Special Programme of Research, Development and Research Training in Human Reproduction (HRP), Department of Sexual, Reproductive, Maternal, Child, Adolescent Health and Ageing, World Health Organization, Geneva, Switzerland; 2 Centro Rosarino de Estudios Perinatales (CREP), Rosario, Argentina; 3 Department of Obstetrics and Gynecology, University of Campinas, Campinas, Brazil; 4 Institut de Recherche en Sciences de la Santé (IRSS), Ouagadougou, Burkina Faso; 5 Department of Public Health-CIGES, Faculty of Medicine, University de La Frontera, Temuco, Chile; 6 School of Public Health, University of Ghana, Accra, Ghana; 7 Aga Khan University, Nairobi, Kenya; 8 Pumwani Maternity Hospital, Pumwani, Kenya; 9 Department of Community Health Sciences, Aga Khan University, Karachi, Pakistan; 10 Department of Pathology and Laboratory Medicine, Aga Khan University, Karachi, Pakistan; 11 University of the Philippines Manila, Manila, Philippines; 12 Institute of Clinical Epidemiology, National Institutes of Health, University of the Philippines Manila, Manila, Philippines; 13 Institute of Child Health and Human Development, National Institutes of Health, University of the Philippines, Manila, Philippines; 14 Research Institute for Tropical Medicine-Department of Health, Manila, Philippines; 15 Department of Vaccines Research, Fundación Para el Fomento de la Investigación Sanitaria y Biomédica de la Comunitat Valenciana (Fisabio), Valencia, Spain; 16 Research Laboratory “Virus, Vectors and Hosts”, LR20IPT02, Pasteur Institute, University Tunis El-Manar (UTM), Tunis, Tunisia; 17 Clinical Investigation Center (CIC2016IPT02), Institut Pasteur de Tunis, University of Tunis El Manar, Tunis, Tunisia; 18 Faculty of Pharmacy, University of Monastir, Monastir, Tunisia

**Keywords:** COVID-19, Omicron, Pregnancy complications, Pregnancy outcomes, Maternal health, Neonatal outcomes, Cohort study

## Abstract

**Background:**

Severe acute respiratory syndrome coronavirus 2 (SARS-CoV-2) infection has raised global concerns regarding its impact on pregnant women and their infants, particularly across evolving variants. This study aimed to determine if infection during pregnancy increased the risk of adverse maternal, perinatal, neonatal, or postpartum outcomes during pre-Omicron and Omicron periods.

**Methods:**

We conducted a prospective cohort study in 10 primarily low- and middle-income countries, comprising public and private maternity hospitals in rural and urban areas. Maternal age and comorbidity adjusted mixed-effects models with country-specific random intercepts were used to estimate relative risks (RRs) comparing participants with and without infection during pregnancy and assess differences by variant period (pre-Omicron vs. Omicron). A total of 16,005 pregnant women were consecutively recruited (all eligible women were enrolled in sequence as they presented at the study facilities) between January 2021 and October 2023 and followed from enrollment through delivery and to six weeks postpartum. Main outcomes included maternal outcomes (haemorrhage, hypertensive disorders of pregnancy, emergency caesarean delivery, maternal death, and modified maternal morbidity and mortality index (MMMI)), and perinatal and neonatal outcomes (preterm birth, congenital anomalies, neonatal intensive care unit (NICU) admission, and severe perinatal morbidity and mortality index (SPMMI)).

**Results:**

Based on reverse transcription polymerase chain reaction (RT-PCR) or antigen (Ag) testing and serial serology, 2,187 participants had confirmed SARS-CoV-2 infection during pregnancy, 116 were classified as probable infection, 7,332 as possible infection, 402 participants were probably uninfected, 1,053 had no evidence of infection, and 4,915 had unknown infection status. During the pre-Omicron era, infected women had higher risks of emergency caesarean delivery (RR 1.26, CI 1.03–1.55); MMMI (RR 1.28, CI 1.13–1.45), preterm birth < 37 weeks (RR 1.73, CI 1.31–2.28), < 34 weeks (RR 3.67, CI 1.92–7.04), < 32 weeks (RR 7.62, CI 2.16–26.85), NICU admission (RR 1.90, CI 1.28–2.83) and SPMMI (RR 1.76, CI 1.01–3.06). During the Omicron era, only preterm birth < 34 weeks remained at significantly elevated risk (RR 1.88, CI 1.04–3.05).

**Conclusions:**

SARS-CoV-2 infection during pregnancy was associated with an increased risk of adverse maternal, perinatal, and neonatal outcomes in the pre-Omicron era, with attenuation during the Omicron period. Persistent elevated risk for preterm birth < 34 weeks underscores the need for ongoing evaluation with emerging variants.

**Supplementary Information:**

The online version contains supplementary material available at 10.1186/s44263-026-00292-9.

## Background

Since the emergence of severe acute respiratory syndrome coronavirus 2 (SARS-CoV-2), concerns about its effects on pregnancy have been raised worldwide. Systematic reviews and meta-analyses summarizing the effect of SARS-CoV-2 infection during pregnancy have demonstrated increased risk for multiple adverse maternal and perinatal outcomes [1, 2]. However, these meta-analyses have been limited by varying study designs, potentially inexact definitions of infection during pregnancy, and differing outcome definitions across included studies.

In addition, most available data have been from earlier in the pandemic. Several studies have shown less severe maternal illness with Omicron variants, yet studies comparing pregnancy-related outcomes in infected and uninfected pregnant women during the Omicron and Omicron sub-variant era remain limited [3–5]. Further, most data were from high-income countries (HICs). Less is known about the impact of infection on pregnancy in low- and middle-income countries (LMICs), where differences in access to higher-level care, underlying comorbidities, and baseline rates of adverse pregnancy outcomes might modify risks [6].

Early in the COVID-19 pandemic, the World Health Organization (WHO) developed a generic protocol for a prospective cohort study among pregnant women to facilitate systematic and harmonized data collection across different settings and to minimize potential biases [7]. The study aimed to determine if SARS-CoV-2 infection during pregnancy increased the risk of adverse maternal, perinatal, neonatal, or postpartum outcomes. The protocol was implemented in 10 diverse, primarily LMICs (Argentina, Brazil, Burkina Faso, Chile, Ghana, Kenya, Pakistan, Philippines, Spain, and Tunisia). We report the pooled results of this study during pre-Omicron and Omicron periods of SARS-CoV-2 variant predominance. Additionally, we report on factors associated with SARS-CoV-2 illness severity, including vaccination, and the association of severity with adverse outcomes among infected women. Detailed evaluation of the effects of COVID-19 vaccination on pregnancy outcomes from this study will be reported separately.

## Methods

This prospective cohort study was conducted between January 2021 and October 2023, across 43 health facilities in the 10 countries (Supplementary Material 1: Table S1). The study design, data collection, and reporting adhered to the STROBE (Strengthening the Reporting of Observational Studies in Epidemiology) guidelines to ensure transparency, completeness, and methodological rigor in the presentation of the research findings. The study protocol, including sample size estimation, is available online [7].

### Study procedures: enrolment

Pregnant or recently pregnant women (within 48 h of delivery or end of pregnancy) attending antenatal care or other obstetric services at participating health facilities were consecutively recruited regardless of SARS-CoV-2 infection status or vaccination status [7]. Only those unable to provide informed consent or assent or to attend follow-up visits were excluded.

At enrolment, all participants had detailed assessment of demographics, past medical and obstetric history, gestational age, details of the current pregnancy including complications and comorbidities, and COVID-19 vaccination status, with review of the obstetric record up to the time of enrolment.

At enrolment, all participants received virologic testing for SARS-CoV-2, either reverse transcription polymerase chain reaction (RT-PCR) or antigen (Ag) testing depending on study site, regardless of symptoms, along with serologic testing for SARS-CoV-2 antibodies [7]. Participants who had never received a COVID-19 vaccine were tested for antibodies against the spike (S) protein of SARS-CoV-2 (Wantai BioPharm SARS-CoV-2 Ab ELISA, China). Vaccinated participants underwent anti-nucleocapsid (N) protein serologic testing (BioRad Platelia SARS-CoV-2 Total Ab Assay, USA) to distinguish infection-induced (S+/N+) from vaccine-acquired antibodies (S+/N-). An exception applied to women who received inactivated whole virus vaccines, which induce production of both anti-S and anti-N antibodies. In these cases, serologic status remained unknown. COVID-19 vaccination status was confirmed using vaccination cards, medical records, or vaccination registries wherever possible. Participants were asked about COVID-19-related symptoms, diagnoses and treatments, and medical and laboratory records were reviewed for COVID-19 diagnoses during and prior to pregnancy.

### Study procedures: follow-up

Follow-up data were collected during regular antenatal care visits, which were typically every 4–6 weeks; however, intervals varied by country. Changes in health status, pregnancy complications, COVID-19 vaccination, COVID-19-related symptoms and laboratory findings since the previous visit were recorded, along with dates. At delivery, data were also collected on mode of delivery and pregnancy outcomes.

Participants presenting with signs or symptoms related to COVID-19 at any time during follow-up received RT-PCR or Ag testing. In addition, all participants who had previous negative SARS-CoV-2 serologic testing at enrolment or follow-up had repeat serologic testing at subsequent follow-up visits including delivery.

Participants were followed through six weeks after delivery, and information was collected on symptoms and health outcomes of both the woman and her neonate(s).

### Definitions

#### Gestational age

Gestational age was estimated based on ultrasound measurement in the first trimester or on last menstrual period (LMP), where start of pregnancy (day 0) was the first day of LMP. If both measurements were available, the best obstetric estimate was used, based on the American College of Obstetricians and Gynecologists algorithm [8].

The first trimester was defined as day 1–97 of pregnancy (week 0–13), second trimester day 98–195 (week 14–27), and third trimester day 196–280+ (week 28–40+).

#### SARS-CoV-2 infection status

**Fig. 1:**
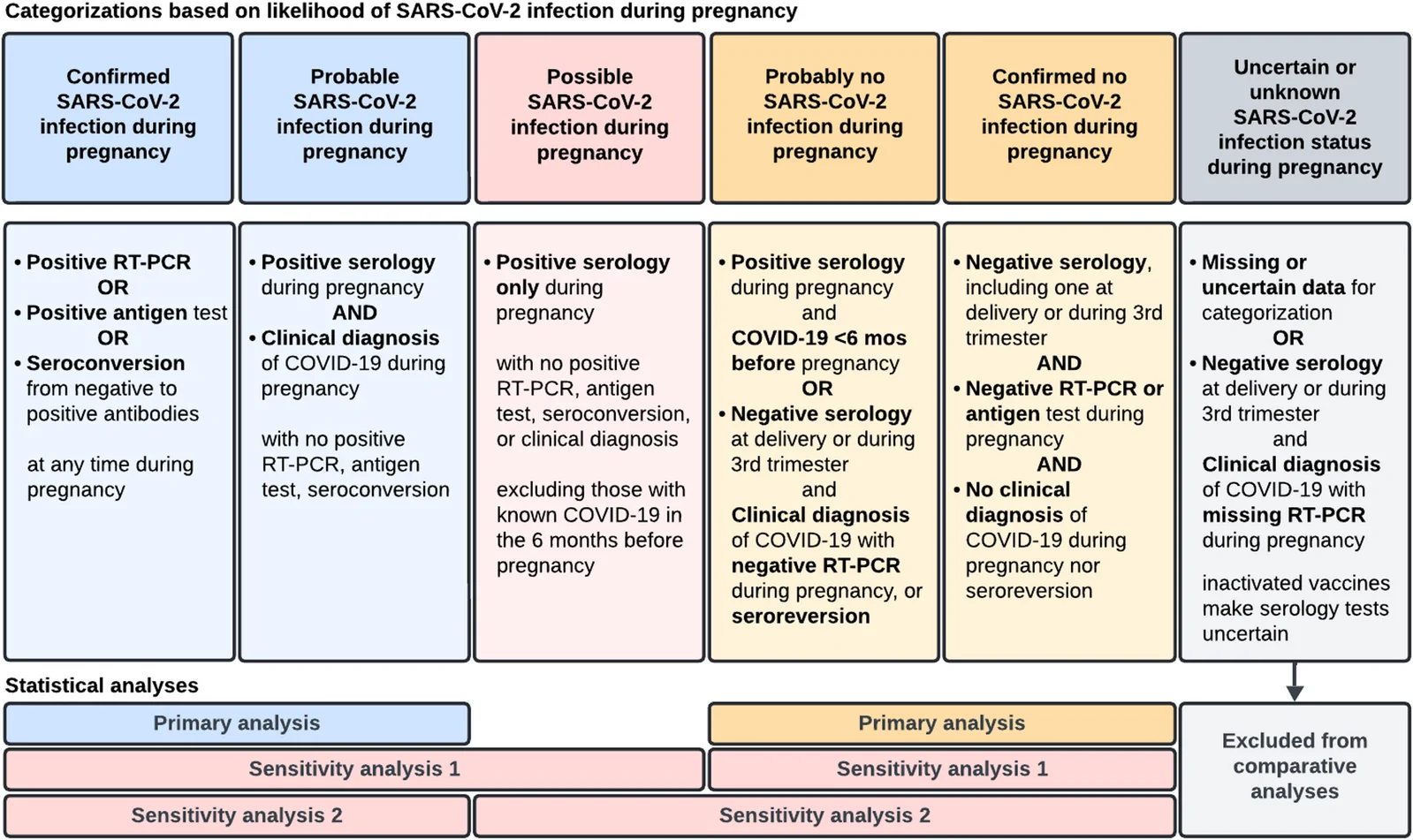
Categorizations based on likelihood of SARS-CoV-2 infection during pregnancy. This figure illustrates the classification of pregnant women according to the likelihood of SARS-CoV-2 infection during pregnancy. Categories were defined based on available diagnostic, clinical, and/or epidemiological evidence, including laboratory confirmation, suspected infection, and no evidence of infection. Criteria used for classification are shown for each category

Women were grouped into six mutually exclusive exposure categories based on the likelihood that SARS-CoV-2 infection occurred during pregnancy: confirmed infected, probably infected, possibly infected, probably uninfected, confirmed uninfected, and unknown (Fig. 1).

Participants were considered to have “confirmed” SARS-CoV-2 infection if they had a positive RT-PCR or Ag test result during pregnancy or seroconversion (not related to vaccination) during follow-up. Those with consistently negative virologic and serologic test results were considered “confirmed uninfected”. Since a positive serologic test might reflect infection prior to pregnancy, most women with positive serology results were categorized as “possibly” infected during pregnancy, unless they also had a clinical diagnosis during pregnancy or documented infection shortly before pregnancy. Participants were classified as having “probable” SARS-CoV-2 infection if they had positive serology and a clinical diagnosis during pregnancy but without virologic confirmation or documented seroconversion. “Probably not infected” included seropositive women who had documented infection within the 6 months before pregnancy, or those with a clinical diagnosis but negative RT-PCR testing and negative serology at the end of pregnancy. Participants whose infection status during pregnancy could not be determined because of missing data or prior receipt of inactivated vaccines were classified as “unknown”.

The date of infection was defined as the date of specimen collection for positive RT-PCR and Ag tests, the midpoint between negative and positive serologic specimen collection for seroconversion, and first day of symptoms for clinical diagnosis. For those with infection, information was collected on symptoms and hospitalization. “Moderate” illness was defined as presence of fever, shortness of breath, or chest pain, while COVID-19-related hospital admission was used as a proxy for “severe” infection. Having at least one symptom but none in the moderate or severe category was defined as “mild” illness.

#### Variant period

Variant of concern (VOC) designation was determined based on country-specific genetic sequencing data from the Global Initiative on Sharing All Influenza Data (GISAID), which had been expanded to include SARS-CoV-2 data [9]. The assigned VOC was the variant predominant in 75% or more of sequenced cases in the participant’s country at the pregnancy start date. Because the timing of variant transitions varied across countries, variant period classification was country-specific rather than based on a single global calendar date. Participants whose pregnancy started during the period of Omicron predominance in their country were classified as ‘Omicron’ and those whose pregnancy started before Omicron predominance were grouped under ‘pre-Omicron’. During the pre-Omicron period, Delta was the predominant variant in most participating countries, with Alpha and Gamma predominating earlier in some settings (Supplementary Material 1: Table S3).

#### Vaccination status

Participants were considered fully vaccinated if they had received a complete primary series (at least one dose for J&J/Janssen or CanSino and at least two doses for all other vaccines); partially vaccinated if they had only received one dose (aside from J&J/Janssen or CanSino); and never vaccinated if they had not received any doses. The “fully vaccinated” group was divided according to whether their most recent dose was during or before pregnancy. Vaccines used across study sites included BNT162b2 (Pfizer–BioNTech, Mainz, Germany / New York, USA), mRNA-1273 (Moderna, Cambridge, MA, USA), CoronaVac (Sinovac Biotech, Beijing, China), BBIBP-CorV (Sinopharm, Beijing Institute of Biological Products, Beijing, China), BBV152 Covaxin (Bharat Biotech, Hyderabad, India), ChAdOx1 nCoV-19 (Oxford–AstraZeneca, Cambridge, UK), AZD1222 (Covishield, Serum Institute of India, Pune, India), Ad26.COV2.S (Johnson & Johnson/Janssen, Beerse, Belgium), Ad5-nCoV (CanSino Biologics, Tianjin, China), and Sputnik (Gamaleya National Research Center for Epidemiology and Microbiology, Moscow, Russia).

### Outcomes

Outcomes were prospectively ascertained using standardized definitions and case report forms, based on clinical documentation from medical records, with data collected at antenatal visits, delivery, and through follow-up to six weeks postpartum.

The following maternal, perinatal, neonatal, and postpartum outcomes were assessed (Supplementary Material 1: Table S2):

#### Maternal and pregnancy outcomes

Miscarriage before 22 weeks of gestation and before 28 weeks of gestation; haemorrhage (antenatal, intrapartum, and immediately postpartum); preeclampsia or eclampsia; hypertensive disorders of pregnancy; thromboembolic disease; preterm labour (< 37 weeks); placental abruption; maternal near-miss indicators at delivery; caesarean delivery; and maternal death (during pregnancy or up to 42 days postpartum). A composite outcome, the modified maternal morbidity and mortality index (MMMI), was defined as the presence of at least one of the following: haemorrhage at labour and delivery, hypertensive disorders of pregnancy (including preeclampsia/eclampsia), thromboembolic disease, preterm labour, placental abruption, any maternal near-miss indicator at delivery, or maternal death.

#### Perinatal and neonatal outcomes

Stillbirth at or after 22 weeks and at or after 28 weeks of gestation; preterm birth (< 37, < 34, and < 32 weeks); low birth weight (< 2500 g) and very low birth weight (< 1500 g); congenital anomalies (at or after 22 weeks and at or after 28 weeks); perinatal death (from 22 weeks gestation to 7 days after birth, and from 28 weeks gestation to 7 days after birth); neonatal death (up to 28 days after birth); and neonatal intensive care unit (NICU) admission. A composite outcome, the severe perinatal morbidity and mortality index (SPMMI), was defined as stillbirth or at least one of the following: hypoxic–ischemic encephalopathy, sepsis, NICU admission for ≥ 7 days, or neonatal death before hospital discharge.

#### Postpartum outcomes

Postpartum haemorrhage, maternal infection or endometritis, and hospital readmission during the postpartum period.

### Data management

Data were collected using paper-based or tablet-based case report forms and subsequently key-entered into a validated web-interface electronic data capture system on the OpenClinica^®^ platform (OpenClinica LLC, Waltham, Massachusetts, USA). Country-specific user accounts were password-protected, with a hierarchy of data access depending on each researcher’s role. Data sent to the server were encrypted using Secure Hash Algorithm 256-bit (SHA256) with Rivest-Shamir-Adleman (RSA) encryption. All study records were anonymised with unique study numbers. A standardised data management plan for data collection, curation, and discrepancy management was coordinated by WHO through web-based dashboards and periodic PDF reports.

### Statistical analysis

Participants with unknown infection status were excluded from both primary and sensitivity analyses. Characteristics of enrolled participants including country, demographics, clinical and obstetric history, SARS-CoV-2 infection status during pregnancy, and VOC designation were summarized using appropriate descriptive statistics. Categorical variables were presented as proportions, while continuous variables were summarized using measures of central tendency (mean or median) and dispersion (standard deviation or quartiles), based on variable distribution. Comparisons of proportions were conducted using Pearson’s chi-squared test or Fisher’s exact test, and comparisons of continuous variables were conducted using the Wilcoxon rank sum test. A p-value < 0.05 was considered statistically significant.

For the primary analysis, participants with confirmed or probable SARS-CoV-2 infection during pregnancy were considered as “SARS-CoV-2 infected”, and those with confirmed or probably no infection during pregnancy were considered as “uninfected” (Fig. 1). Risk of adverse pregnancy outcomes related to SARS-CoV-2 infection during pregnancy were evaluated by comparing the absolute risk of outcomes in the infected group to the uninfected group and by estimating the relative risk (RR) using log-binomial generalized linear mixed models (GLMMs). We reviewed country-specific forest plots for each outcome and VOC period. Countries with the most extreme RR estimates (highest or lowest) were excluded one at a time, and models were refitted to evaluate whether any single country disproportionately influenced the pooled estimate. All models were adjusted for maternal age at enrollment and presence of at least one comorbidity. Additional covariates, including education level, gravidity, and facility level, were evaluated during model development but were not retained in the final models as their inclusion led to convergence failures, particularly for less common outcomes and in variant-stratified analyses. Country-specific random intercepts were used to account for unmeasured between-country differences, including those related to these factors. Modification of effects by VOC was evaluated by fitting the model with an interaction term between SARS-CoV-2 infection and VOC period, and RR estimates were reported for combined sample and stratified for the pre-Omicron and Omicron periods. For all estimates, 95% confidence intervals (CI) were provided.

In sensitivity analyses, these models were repeated twice: first by adding the “possibly infected” during pregnancy group to the “infected” group and second, by adding the “possibly infected” group to the “uninfected” group.

For each adverse pregnancy outcome of interest, denominators were established based on contribution to the gestational period at risk and timing of enrolment in the study, as some outcomes such as miscarriage occur only within specific time periods (Supplementary Material 1: Table S2). All analyses incorporated restrictions to ensure temporal plausibility, i.e., that only outcomes occurring after the date of SARS-CoV-2 infection were considered in the infected group; outcomes occurring before the infection date were recoded as non-events. For multiple pregnancies, neonatal outcomes were assessed using the worst-case outcome among neonates from the same pregnancy. Those who were lost to follow up prior to completion of pregnancy and those whose SARS-CoV-2 testing occurred more than 48 h after delivery were excluded. For preterm birth, an additional subgroup analysis was conducted. In the main analysis, the sample was restricted to participants with positive SARS-CoV-2 tests occurring prior to the gestation week for the preterm birth outcome being considered but otherwise included in all live births. For the subgroup analyses, further restrictions were applied: only participants with (1) enrollment dates, or (2) negative SARS-CoV-2 testing dates, prior to the gestation week were considered ensuring temporal alignment between infection status and outcome risk. For congenital anomalies, a post-hoc analysis was conducted among infected women, modelling the RR of congenital anomaly by trimester of infection, adjusting for maternal age and comorbidity with country-level clustering.

To explore determinants of illness severity within the SARS-CoV-2 infected subgroup, a mixed effects logistic regression model, using country-specific random intercepts, was fitted to evaluate the association between maternal age, underlying conditions, vaccination status and VOC period with illness severity. Odds ratios (ORs) for moderate or severe illness compared to asymptomatic or mild infection and 95% CI were estimated. The risk of pregnancy, perinatal, and neonatal outcomes in the moderate or severe illness group relative to the asymptomatic or mild group, controlling for maternal age and presence of underlying conditions, were assessed using the GLMM model as above.

Analyses were conducted using R version 4.4.2 (R Foundation for Statistical Computing, Vienna, Austria).

## Results

### Study population

**Fig. 2:**
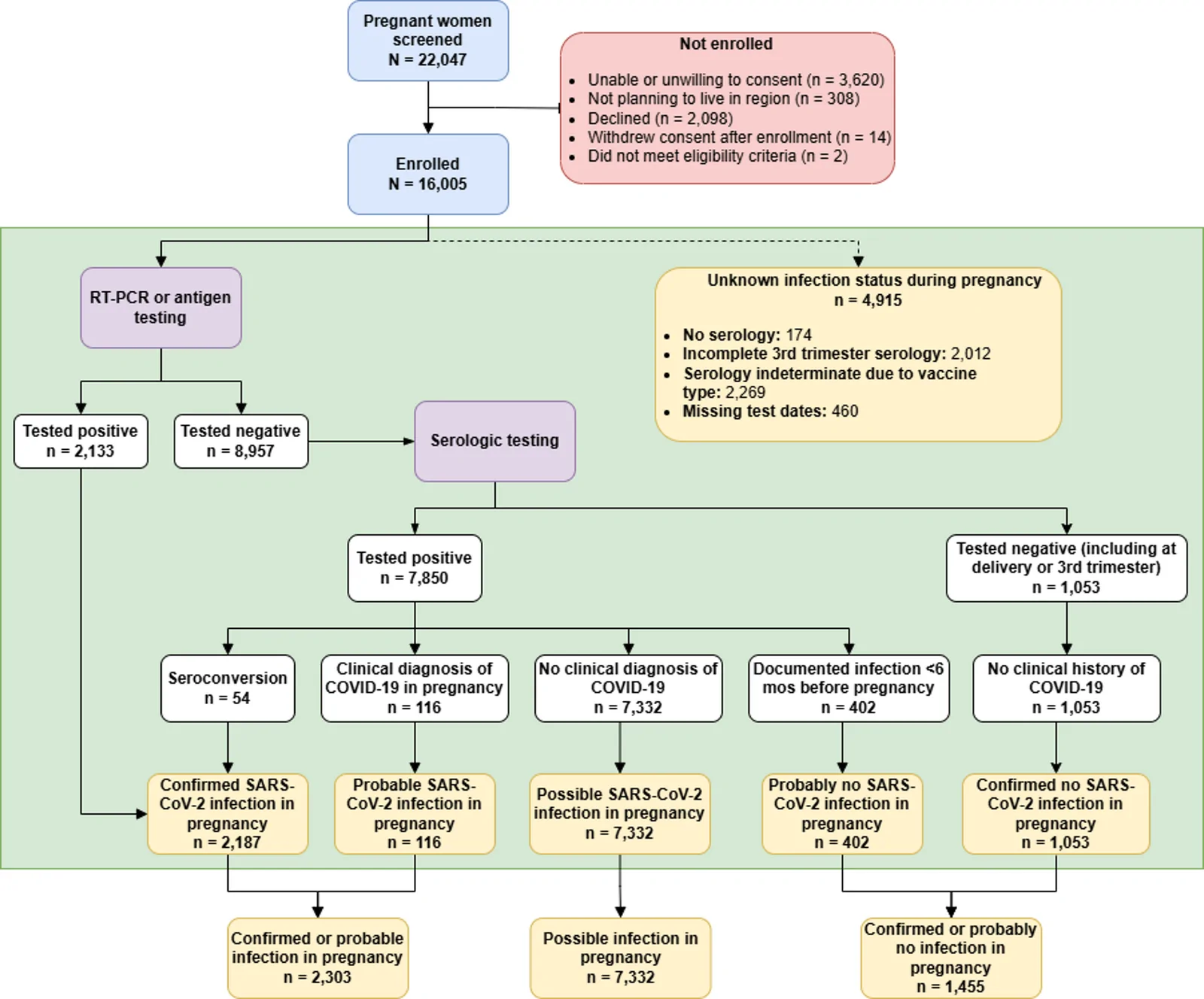
Study flowchart and algorithm for determining infection status during pregnancy. This figure presents the study flowchart, including participant enrollment and inclusion in the analysis, along with the algorithm used to determine SARS-CoV-2 infection status during pregnancy. The algorithm incorporates laboratory results, clinical diagnoses, and timing of infection relative to pregnancy to assign infection status. Numbers of participants at each stage of the study are shown

Of 22,047 pregnant women screened at 43 facilities in 10 countries, 16,005 (72.6%) were enrolled. Study flow, assignment to infection status groups, and final analytic samples are shown in Fig. 2.

Based on laboratory testing and clinical diagnoses over the study, 2,187 (13.7%) participants were classified as having confirmed SARS-CoV-2 infection during pregnancy, 116 (0.7%) had probable infection, 7,332 (45.8%) had possible infection, 402 (2.5%) probably had no infection, 1,053 (6.6%) had confirmed no infection during pregnancy, and 4,915 had unknown infection status.

The distribution of participants by SARS-CoV-2 infection status across the participating countries is presented in Supplementary Material 1: Table S4. Most enrolled participants began their pregnancies in 2021 or 2022 (Supplementary Material 1: Fig S1). Of 15,912 with available data, 11,845 pregnancies (74.4%) began before the period of Omicron predominance (pre-Omicron) and 4,067 (25.6%) began during the period of Omicron predominance (Supplementary Material 1: Fig S2). Most infections (80.8%) also occurred in the pre-Omicron period. Among 2,233 infected women with data, 338 (15.1%) were infected in the first trimester, 585 (26.2%) in the second, and 1,310 (58.7%) in the third (median gestational age at infection: 30.3 weeks [Interquartile range (IQR) 18.1–37.7].

Women infected with SARS-CoV-2 during pregnancy were more likely than uninfected women to be multigravida (73.5% vs. 69.0%, *p* < 0.004), single or previously married (24.3% vs. 13.5%, *p* < 0.001), to have higher education (41.8% vs. 36.6%, *p* < 0.001), and to be unemployed or working in informal jobs (57.5% vs. 45.4%, *p* < 0.001) (Table 1). Both groups were similar with respect to age at enrollment and alcohol consumption. Although the median gestational age at enrollment was 36 weeks for both groups, a higher proportion of infected women were enrolled earlier in pregnancy (6.4% vs. 4.3% before 14 weeks, 22.0% vs. 10.7% between 14 and 27 weeks, 71.6% vs. 85.0% after 27 weeks, *p* < 0.001). Most study participants were seen in tertiary care hospitals, more commonly among those with infection (94.7% vs. 80.4%, *p* < 0.001).

**Table 1:** Selected participant characteristics by SARS-CoV-2 infection status during pregnancy

Characteristic	Confirmed or probably infected *N* = 2303^*1*^	Confirmed or probably uninfected *N* = 1455^*1*^	Unadjusted *p*-value^2^
**Maternal age at enrollment**			
**In years**^**1**^:	30 (26, 35)	31 (27, 35)	0.027
**In years by groups**:			0.256
<20	67 (2.9)	36 (2.5)	
20–35	1,712 (74.9)	1,060 (73.2)	
>=36	505 (22.1)	352 (24.3)	
Missing/Unknown	19	7	
**Gestational age at enrollment**			
**In weeks**^**1**^:	36 (26, 39)	36 (30, 38)	0.466
**In weeks by group**:			< 0.001
<14 weeks	147 (6.4)	63 (4.3)	
14–27 weeks	505 (22.0)	155 (10.7)	
> 27 weeks	1,644 (71.6)	1,235 (85.0)	
Missing/Unknown	7	2	
**Enrolled in the 48 h after delivery**			0.034
No	2,056 (89.3)	1,265 (86.9)	
Yes	247 (10.7)	190 (13.1)	
**Type of facility**			< 0.001
Primary	77 (3.5)	167 (12.1)	
Secondary	41 (1.8)	105 (7.6)	
Tertiary	2,107 (94.7)	1,113 (80.4)	
Missing/Unknown	78	70	
**Number of pregnancies (including current)**			0.004
Primigravida	607 (26.5)	449 (31.0)	
Multigravida	1680 (73.5)	1000 (69.0)	
Missing/Unknown	16	6	
**Marital status**			0.001
Single or Previously Married	553 (24.3)	195 (13.5)	
Married/Cohabiting	1720 (75.7)	1248 (86.5)	
Missing/Unknown	30	12	
**Highest level of education completed**			0.001
Basic Education	472 (21.4)	416 (29.0)	
Secondary Education	809 (36.8)	493 (34.4)	
Higher Education	919 (41.8)	524 (36.6)	
Missing/Unknown	103	22	
**Occupation**			< 0.001
Employed or Self-employed	963 (41.8)	789 (54.2)	
Unemployed or Informal or Other	1,325 (57.5)	660 (45.4)	
Missing/Unknown	15	6	
**Alcohol consumption within the past 30 days**			0.066
Yes	50 (2.2)	19 (1.3)	
No	2,209 (97.8)	1,423 (98.7)	
Missing/Unknown	44	13	
**Currently smoke any tobacco products**			< 0.001
Yes	41 (1.8)	24 (1.7)	
No	2,211 (96.0)	1,421 (98.3)	
Missing/Unknown	51	10	
**Presence of medical conditions** ^**§**^			0.099
0	1,604 (70.5)	2,597 (69.7)	
1	475 (20.9)	816 (21.9)	
2 or more	142 (6.2)	234 (6.3)	
Missing/Unknown	27	6	
Vaccination status*			< 0.001
Never	753 (35.5)	630 (45.5)	
Partially	169 (8.0)	162 (11.7)	
Fully, last dose before pregnancy	502 (23.7)	201 (14.5)	
Fully, last dose during pregnancy	697 (32.9)	393 (28.4)	
Missing/Unknown	182	69	

^1^ Median (IQR); n (%)

^2^ Pearson’s Chi-squared test; Wilcoxon rank sum test; Pearson’s Chi-squared test

§ Number of conditions counts the simultaneous presence of the following: heart disease, lung disease, kidney disease, liver disease, hypertension, type 1 or type 2 diabetes, HIV, tuberculosis, Body Mass Index (BMI) ≥ 30, and BMI < 18.5. *Vaccination status represents the status at the end of follow-up, rather than baseline, so vaccination could occur after infection

Conditions such as heart disease, lung disease, hypertension, and diabetes were consistently more prevalent in the infected group; however, neither individual conditions nor the overall number of conditions differed significantly between groups (Supplementary Material 1: Table S5). Vaccination status by the end of the study (potentially occurring after infection) differed significantly between both groups. Infected participants were less likely to have never been vaccinated (35.3% vs. 45.5%) and more likely to be fully vaccinated before pregnancy (23.7% vs. 14.5%) (Table 1). Participants receiving inactivated vaccines were classified as having unknown infection status unless they had a positive virologic test, which affected the proportion vaccinated across all infection groups (Supplementary Material 1: Table S6).

### Maternal and pregnancy outcomes.

Of the participants included in the analytic sample, 604 (5.4%) participants were lost to follow up before end of pregnancy, leaving 2,244 participants with confirmed or probable infection and 1,390 participants with confirmed or probably no infection. Participants classified as possibly infected (*n* = 6,852) were considered in the sensitivity analysis.

Accounting for variation by country and after adjusting for maternal age and presence of at least one comorbidity, the risks of emergency caesarean delivery (RR 1.26, CI 1.03–1.55) and the composite outcome of maternal morbidity and mortality according to the modified MMMI (RR 1.28, CI 1.13–1.45) were significantly higher among women infected with SARS-CoV-2 compared to the uninfected group during the pre-Omicron period (Table 2 and Supplementary Material 1: Fig S3). During the Omicron era, RR estimates for both outcomes were no longer significantly elevated, and the risk for emergency caesarean delivery was significantly lower among women infected with SARS-CoV-2. None of the other maternal outcomes evaluated had significantly elevated RRs in either variant period **(**Table 2**)**.

**Table 2:** Maternal, pregnancy, perinatal, neonatal and postpartum outcomes by SARS-CoV-2 infection status during pregnancy^¥^

Outcome^§^	Confirmed or probably infected^£^ *N* = 2,305^*1*^	Confirmed or probably uninfected^£^ *N* = 1,455^*1*^	Adjusted infected vs. uninfected random-effects model Relative Risk (95% Confidence Interval); *p*-value^2^		
			Overall sample (Events / Total)	Restricted to pre-Omicron (Events / Total)	Restricted to Omicron (Events/Total)
**MATERNAL AND PREGNANCY OUTCOMES**					
Miscarriage (before 22 weeks of gestation)*			16/401	6/212	10/189
Yes	11 (3.7)	5 (4.9)	0.69 (0.42,1.13) *p* = 0.1393	0.86 (0.11,7.09) *p* = 0.8882	0.76 (0.52,1.11) *p* = 0.1538
No	290 (96.4)	97 (95.1)			
At risk	301	102			
Miscarriage (before 28 weeks) *			16/403	6/213	10/190
Yes	11 (3.6)	5 (4.9)	0.69 (0.42,1.13) *p* = 0.1362	0.86 (0.11,7.07) p = (0.8882)	0.76 (0.52,1.11) *p* = 0.1512
No	292 (96.4)	97 (95.1)			
At risk	303	102			
**Haemorrhage (antepartum**,** intrapartum**,** or immediately postpartum)**			114/3,576	90/2,956	24/620
Yes	79 (3.6)	36 (2.6)	1.64 (0.66,4.04) *p* = 0.2869	2.11 (0.98,4.51) *p* = 0.0553	0.51 (0.23,1.12) *p* = 0.0927
No	2,140 (96.4)	1,344 (97.4)			
At risk	2,219	1,380			
**Preeclampsia/eclampsia**			240/3,605	185/2971	55/634
Yes	156 (7.0)	84 (6.1)	1.32 (0.77,2.26) *p* = 0.3131	1.46 (0.80,2.68) *p* = 0.2213	0.93 (0.63,1.38) *p* = 0.7099
No	2,085 (93.0)	1,305 (93.9)			
At risk	2,241	1,389			
**Hypertensive disorders of pregnancy**			327/3,606	244/2,972	83/634
Yes	218 (9.7)	109 (7.9)	1.39 (0.83,2.33) *p* = 0.2078	1.60 (0.94,2.74) *p* = 0.0849	0.93 (0.59,1.45) *p* = 0.7422
No	2,024 (90.3)	1,280 (92.2)			
At risk	2,242	1,389			
**Thromboembolic disease**			7/3,605	7/2,971	0/634
Yes	4 (0.2)	3 (0.2)	0.84 (0.21,3.30) *p* = 0.8003	0.89 (0.22,3.54) *p* = 0.8706	—
No	2,236 (99.8)	1,385 (99.8)			
At risk	2,240	1,388			
**Preterm labour**			178/2,355	131/1,855	47/500
Yes	118 (7.5)	60 (7.6)	0.90 (0.67,1.22) *p* = 0.5063	0.79 (0.58,1.08) *p* = 0.1411	1.27 (0.72,2.23) *p* = 0.4072
No	1,459 (92.5)	731 (92.4)			
At risk	1,577	791			
**Placental abruption**			17/3,605	14/2,971	3/634
Yes	10 (0.5)	7 (0.5)	0.93 (0.39,2.25) *p* = 0.8749	0.91 (0.36,2.35) *p* = 0.8498	0.82 (0.22,3.13) *p* = 0.7745
No	2,230 (99.6)	1,381 (99.5)			
At risk	2,240	1,388			
**Maternal near-miss at delivery**			65/3532	58/2908	7/624
Yes	36 (1.7)	29 (2.1)	1.14 (0.57,2.28) *p* = 0.7044	1.25 (0.65,2.43) *p* = 0.5043	0.59 (0.13,2.70) *p* = 0.4943
No	2,143 (98.4)	1,343 (97.9)			
At risk	2,179	1,372			
**Caesarean delivery**			766/3,550	635/2,936	131/614
Emergency	506 (23.0)	265 (19.3)	1.17 (0.93,1.47) *p* = 0.1908	1.26 (1.03,1.55) *p* = 0.0267	0.73 (0.56,0.94) *p* = 0.0156
Other delivery modes^α^	1,694 (77.0)	1,105 (80.7)			
At risk	2,200	1,370			
**Maternal death**			9/3,601	7/2,967	2/634
Yes	8 (0.4)	1 (0.1)	3.52 (0.92,13.52) *p* = 0.0664	3.23 (0.75,13.96) *p* = 0.1169	—
No	2,230 (99.6)	1,387 (99.9)			
At risk	2,230	1,388			
**Modified maternal morbidity and mortality index (MMMI)**			746/3,558	572/2,928	174/630
Yes	465 (21.1)	282 (20.5)	1.14 (0.94,1.37) *p* = 0.747	1.28 (1.13,1.45) *p* = 0.0001	0.76 (0.48,1.22) *p* = 0.2612
No	1,741 (78.9)	1,094 (79.5)			
At risk	2,206	1,376			
**PERINATAL AND NEONATAL OUTCOMES**					
**Stillbirth at/after 22 weeks**			21/3,573	19/2,956	2/617
Yes	15 (0.7)	7 (0.5)	1.36 (0.52, 3.60) *p* = 0.5344	1.26 (0.47,3.39) *p* = 0.6487	—
No	2,203 (99.3)	1,374 (99.5)			
At risk	2,218	1,381			
**Stillbirth at/after 28 weeks**			20/3,558	18/2,943	2/615
Yes	14 (0.6)	7 (0.5)	1.24 (0.47,3.25) *p* = 0.6624	1.12 (0.42,2.94) *p* = 0.8241	—
No	2,190 (99.4)	1,373 (99.5)			
At risk	2,204	1,380			
**Preterm birth (< 37 weeks)**			540/2,903	422/2,380	118/523
Yes	361 (23.4)	181 (13.2)	1.53 (1.26,1.84) p = < 0.0001	1.73 (1.31,2.28) *p* = 0.0001	1.02 (0.68,1.53) *p* = 0.9102
No	1,181 (76.6)	1,192 (86.8)			
At risk	1,542	1,373			
**Preterm birth (< 34 weeks)**			120/2,655	93/2,162	27/493
Yes	95 (7.4)	26 (1.9)	3.01 (1.74,5.21) *p* = 0.0001	3.67 (1.92,7.04) *p* = 0.0001	1.81 (1.07,3.05) *p* = 0.0272
No	1,195 (92.6)	1,347 (98.1)			
At risk	1,290	1,373			
**Preterm birth (< 32 weeks)**			58/2,539	43/2,061	15/478
Yes	50 (4.3)	9 (0.7)	4.56 (1.50,13.81) *p* = 0.0074	7.62 (2.16,26.85) *p* = 0.0016	1.62 (0.70,3.72) *p* = 0.2584
No	1,123 (95.7)	1,364 (99.3)			
At risk	1,173	1,373			
**Low birth weight (< 2**,**500 g)**			454/3,195	369/2,623	85/572
Yes	286 (15.1)	171 (13.0)	1.16 (0.92, 1.46) *p* = 0.2078	1.19 (0.92,1.53) *p* = 0.1817	0.96 (0.68,1.36) *p* = 0.8153
No	1,607 (84.8)	1,141 (87.0)			
At risk	1,893	1,312			
**Very low birth weight (< 1**,**500 g)**			88/3,195	76/2,623	12/572
Yes	51 (2.7)	38 (2.9)	1.10 (0.52,2.33) *p* = 0.7972	1.20 (0.50,2.87) *p* = 0.6832	0.72 (0.48,1.06) *p* = 0.0960
No	1,842 (97.3)	1,274 (97.1)			
At risk	1,893	1,312			
**Congenital anomaly (at or after 22 weeks)**			87/2,753	76/2,344	11/409
Yes	63 (3.7)	24 (2.3)	2.06 (0.94,4.50) *p* = 0.0697	2.27 (0.80,6.44) *p* = 0.1232	0.75 (0.38,1.47) *p* = 0.4028
No	1,645 (96.3)	1,032 (97.7)			
At risk	1,708	1,056			
**Congenital anomaly (at or after 28 weeks)**			83/2,741	73/2,334	10/407
Yes	59 (3.5)	24 (2.3)	1.90 (0.94,3.83) *p* = 0.0724	2.10 (0.83,5.36) *p* = 0.1178	0.73 (0.41,1.31) *p* = 0.2940
No	1,637 (96.5)	1,032 (97.7)			
At risk	1,696	1,056			
**Perinatal death (22-weeks stillbirths + early neonatal deaths)**			41/3,573	38/2,956	3/617
Yes	27 (1.2)	15 (1.1)	1.25 (0.77,2.02) *p* = 0.3659	1.37 (0.86,2.18) *p* = 0.1853	0.87 (0.25,3.01) *p* = 0.8307
No	2,191 (98.8)	1,366 (98.9)			
At risk	2,218	1,381			
**Perinatal death (28-weeks stillbirths + early neonatal deaths)**			37/3,558	34/2,943	3/615
Yes	24 (1.1)	14 (1.0)	1.21 (0.79,1.86) *p* = 0.3867	1.33 (0.88,2.0) *p* = 0.1711	0.88 (0.25,3.03) *p* = 0.8363
No	2,180 (98.9)	1,366 (98.9)			
At risk	2,204	1,380			
**Neonatal/infant death**			32/3,509	30/2,896	2/613
Yes	20 (0.9)	13 (1.0)	0.92 (0.26,3.33) *p* = 0.9022	1.16 (0.38,3.55) *p* = 0.7984	—
No	2,152 (99.1)	1,346 (99.0)			
At risk	2,172	1,359			
**Neonatal Intensive Care Unit (NICU) admission**			468/3,495	383/2,884	85/611
Yes	321 (14.9)	150 (11.1)	1.63 (1.06,2.51) *p* = 0.0269	1.90 (1.28,2.83) *p* = 0.0016	0.83 (0.55,1.26) *p* = 0.3889
No	1,840 (85.2)	1,206 (88.9)			
At risk	2,161	1,356			
**Severe Perinatal Morbidity and Mortality Index (SPMMI)**			281/3,502	230/2,898	51/604
Yes	196 (9.1)	89 (6.5)	1.57 (0.97,2.54) *p* = 0.0688	1.76 (1.01,3.07) *p* = 0.0456	0.87 (0.47,1.61) *p* = 0.6527
No	1,967 (91.0)	1,273 (93.5)			
At risk	2,163	1,362			
**POSTPARTUM OUTCOMES**					
**Postpartum haemorrhage**			30/3,357	26/2,756	4/601
Yes	12 (0.6)	18 (1.4)	0.51 (0.23,1.09) *p* = 0.0819	0.36 (0.16,0.83) *p* = 0.0169	—
No	2,060 (99.4)	1,291 (98.6)			
At risk	2,072	1,309			
**Postpartum complications: Maternal infection or endometritis**			77/3,332	57/2,745	20/587
Yes	53 (2.6)	25 (1.9)	1.35 (1.04,1.74) *p* = 0.0236	1.39 (0.94,2.05) *p* = 0.0957	1.20 (0.71,2.02) *p* = 0.5013
No	1,998 (97.4)	1,278 (98.1)			
At risk	2,051	1,303			
**Any readmission in the postpartum period**			55/3,348	43/2,759	12/589
Yes	34 (1.7)	21 (1.6)	1.09 (0.66,1.80) *p* = 0.7328	0.98 (0.53,1.78) *p* = 0.9339	1.72 (0.42,7.08) *p* = 0.4538
No	2,032 (98.4)	1,283 (98.4)			
At risk	2,066	1,304			

^1^ n (%)

^2^ Pearson’s Chi-squared test

*Denominator: All completed pregnancies enrolled at < 20 weeks of gestation

^¥^ N appears at multiple rows to reflect change in the denominator due to difference in outcome definitions

§ M/U = Missing or Unknown

£ N (%)

α: other delivery modes include spontaneous vaginal delivery, instrumental delivery, and planned caesarean delivery

Some outcomes could not be evaluated because of small numbers or lack of model convergence

Eight maternal deaths were recorded out of 2,238 (0.4%) SARS-CoV-2-infected women (3 due to confirmed COVID-19, 1 due to postpartum abdominal infection, and 4 unspecified), and 1 out of 1,388 (0.1%) non-infected women (due to pre-eclampsia). Of these nine deaths, 7 occurred during the pre-Omicron era and 2 during the Omicron era.

### Perinatal and neonatal outcomes

During the pre-Omicron period, the adjusted risks of preterm birth < 37 weeks (RR 1.73, CI 1.31–2.28), as well as < 34 weeks (RR 3.67, CI 1.92–7.04) and < 32 weeks (RR 7.62, CI 2.16–26.85) were significantly higher in the infected group compared to the uninfected group. During the Omicron period, which had a smaller number of participants, the only RR that remained significantly elevated was for preterm birth < 34 weeks (RR 1.81, CI 1.07–3.05). The RR point estimate for preterm birth < 32 weeks was similar to that for 34 weeks; however, this association was not statistically significant (RR 1.62, CI 0.70–3.71) (Table 2 and Supplementary Material 1: Fig S3). In the first subgroup analyses for preterm birth, which was restricted to those with enrolment dates prior to the respective gestation week, RRs were slightly attenuated but remained significant for preterm birth at all three gestation ages in the pre-Omicron era (Supplementary Material 1: Table S7). The significantly increased RR for preterm birth < 34 weeks observed in the Omicron era for the main analysis was no longer observed. In the second subgroup analysis based on dates of negative testing, no significant RRs were observed.

The risk of NICU admission was significantly higher in the infected group during the pre-Omicron period (RR 1.90, CI 1.28–2.83), as was the risk of SPMMI (RR 1.76, CI 1.01–3.06), but not during the Omicron period. No significant differences were found between groups for other perinatal and neonatal outcomes evaluated.

### Postpartum outcomes

The risk of postpartum complications was significantly higher among infected women overall (RR 1.35 CI 1.04–1.74). During the pre-Omicron period, women with SARS-CoV-2 infection had a significantly lower risk of hemorrhage within the 42 days after delivery compared to the uninfected group (RR 0.36, CI 0.16–0.83) (Table 2 and Supplementary Material 1: Fig S3).

### Sensitivity Analysis

Results for the first sensitivity analysis, which combined “possibly infected” women with the “infected” group, are shown in Supplementary Material 1: Tables S8 - S10. In this analysis, RR point estimates for most outcomes were similar to those in the main analysis or attenuated, with some outcomes no longer significant (e.g., emergency caesarean delivery and MMMI) and others becoming significant despite similar RRs (e.g., pre-eclampsia/eclampsia and hypertensive disorders or pregnancy). The only exception to this was for stillbirth and perinatal death, for which RR estimates were higher relative to the main analysis in the pre-Omicron period (e.g., for perinatal death from 28 weeks, the RR was 2.06 (CI 1.45–2.92)). Due to small numbers, these outcomes could not be evaluated during the Omicron period.

In the first sensitivity analysis, the only outcomes with significantly elevated RRs for the infected group in the Omicron period were preterm birth < 34 weeks (RR 2.17, CI 1.46–3.33) and preterm birth < 32 weeks (RR 1.38, CI 1.07–1.78). Risks for congenital anomalies were notably lower (RR 0.39, CI 0.18–0.84 after 28 weeks).

Results of the second sensitivity analysis, including women “possibly infected” during pregnancy in the uninfected group, are shown in Supplementary Material 1: Tables S11 - S13. Overall, notable differences with the main analysis include an increased risk for maternal hemorrhage for those in the infected group in the pre-Omicron era (RR 2.46, CI 1.90,3.18) and decreased risks of miscarriage (RR 0.73, CI 0.55–0.97; Omicron) and of stillbirth (RR 0.55, CI 0.36–0.82 for 28 weeks; pre-Omicron). In this second sensitivity analysis, the only outcomes with significantly elevated RRs for the infected group in the Omicron period were congenital anomalies (RR 1.95, CI 1.02–3.72 after 28 weeks). In the post-hoc analysis of congenital anomalies among infected women, first-trimester infection was associated with higher risk compared to third-trimester infection (RR 1.65 [95% CI 1.08–2.53], *p* = 0.022).

### Severity of SARS-CoV-2 infections and related outcomes

Data on SARS-CoV-2 infection-related symptoms were available for 2,260 (98.1%) women with infection. Of these, 1938 (85.8%) were asymptomatic or had mild symptoms and 322 (14.2%) had moderate to severe symptoms. The most frequently reported symptoms during the pre-Omicron era were fever (37.2%), cough (28.9%) and muscle pain (28.5%) whereas runny nose (27.9%), headache (26.7%) and fever (26.6%) were most frequent during the Omicron period (Supplementary Material 1: Fig S4).

Additionally, the prevalence of moderate to severe infection was significantly lower during the Omicron period compared to the pre-Omicron period (8.9% vs. 15.5%), with an adjusted OR of 0.53 (CI 0.35–0.80), after adjusting for maternal age, comorbidities, and vaccination status (Table 3). Compared to individuals who were never vaccinated, those who were partially vaccinated had 41% lower odds (OR 0.59; CI 0.36–0.97) and those fully vaccinated during pregnancy had 36% lower odds (OR = 0.64, 95% CI: 0.44–0.95) of experiencing moderate or severe illness. Fully vaccinated individuals whose last dose was before pregnancy showed no significant reduction in risk (OR = 0.78, 95% CI: 0.52–1.15).

**Table 3:** Regression model to determine factors associated with severity of illness among SARS-CoV-2 infected women

Covariates in the model	Summary Statistics by Symptoms		Odds Ratio of Moderate or Severe Symptoms	
	**Asymptomatic or Mild**	**Moderate or Severe**	**Odds Ratio OR 95% Confidence Interval** ^1,^	**p-value**
	*N* = 1,938	*N* = 322		
**Maternal age at enrollment (years)**				
Median [Q1, Q3]	30 [26,35]	31 [27,35]	1.03 (0.90,1.18)	0.654
Missing/Unknown	19	0		
**Presence of medical conditions**				
0	1,383 (87.4%)	200 (12.6%)	—	
1 or more conditions	482 (80.9%)	114 (19.1%)	1.95 (1.46,2.60)	< 0.001
Missing/Unknown	73	8		
**Vaccination status**				
Never vaccinated	616 (82.2%)	133 (17.8%)	—	
Partially vaccinated	145 (86.3%)	23 (13.7%)	0.59 (0.36,0.97)	0.038
Fully vaccinated, last dose before pregnancy	419 (86.7%)	64 (13.3%)	0.78 (0.52,1.15)	0.212
Fully vaccinated, last dose during pregnancy	591 (86.8%)	90 (13.2%)	0.64 (0.44,0.95)	0.026
Missing/Unknown	167	12		
**Variant of concern**				
pre-Omicron	1,548 (84.5%)	284 (15.5%)	—	
Omicron	390 (91.1%)	38 (8.9%)	0.53 (0.35,0.80)	0.003

^1^ Odds Ratios determine association after adjusting for other covariates in the model

£: Maternal age OR per 5-year increase

Pregnant women who experienced moderate to severe illness were at significantly increased risk of a near-miss indicator at delivery (RR 3.42, CI 2.18–5.36) and caesarean delivery (RR 1.33, CI 1.05–1.69), with borderline significance for MMMI (RR 1.24, CI 0.99–1.69) (for all participants; numbers were too small to evaluate separately by variant) (Supplementary Material 1: Table S14). 

## Discussion

In this large multi-country prospective cohort study, conducted primarily in LMICs, we evaluated whether SARS-CoV-2 infection during pregnancy increases the risk of adverse maternal, perinatal, neonatal, or postpartum outcomes compared with women without infection during pregnancy, in both pre-Omicron and Omicron periods of SARS-CoV-2 variant predominance. We found that the risks of several adverse outcomes, such as emergency caesarean delivery, MMMI, preterm birth, NICU admission, SPMMI and postpartum hemorrhage were significantly increased following SARS-CoV-2 infection in pregnancy during the pre-Omicron era. However, these risks were no longer significantly elevated during the Omicron period, for all outcomes except preterm birth before 34 weeks of gestation. Continued evaluation of SARS-CoV-2 infection and pregnancy outcomes, particularly in the context of emerging variants and Omicron subvariants, as well as further investigation of the effects of COVID-19 vaccination during these periods, is warranted.

A strength of our study was its prospective design, which used rigorous definitions for both exposure and outcomes. We determined SARS-CoV-2 infection status during pregnancy through universal virologic testing and serial serologic assessments. In contrast, previous individual studies and meta-analyses on this topic have been limited by differing and inexact definitions of exposure status, e.g., relying on a single virologic test to classify participants as “negative”, increasing the risk of misclassification bias. Others preferentially identified cases with symptomatic infection or at delivery, potentially skewing results toward those more likely to experience adverse outcomes [1, 2]. Another strength of our study was the inclusion of a large sample of participants from LMICs in distinct parts of the world. Most prior studies on COVID-19 and pregnancy have been conducted in HICs, where greater access to higher-level care could improve illness-related outcomes while potentially increasing certain clinician-initiated interventions, such as emergency caesarean delivery [10]. Conversely, in many LMICs, higher baseline rates of outcomes like preterm birth can make it more challenging to detect small absolute increases associated with SARS-CoV-2 infection [11]. Differences in underlying comorbidities or in exposures due to socioeconomic factors in LMICs could also modify risk. Finally, our study is among the few that have evaluated RRs of adverse pregnancy outcomes by comparing pregnant women with and without SARS-CoV-2 infection during the Omicron period. This provides a more accurate reflection of current risks than data collected earlier in the pandemic [3–5].

A main limitation of our study was that it was originally designed and powered to incorporate serologic testing at a time when few women had previous infection, so the presence of antibody would reflect recent infection [7]. However, by the time the study started in many settings, most women were already seropositive. Additionally, data had also emerged that SARS-CoV-2 antibodies could persist over 12 months, meaning that seropositivity could reflect infection prior to pregnancy [12]. Excluding participants whose infection status was not confirmed or probable from the primary analysis improved the accuracy of comparisons, but it also reduced our effective sample size and made our study sample less representative of the general antenatal population, especially as the pandemic progressed. Given the loss of statistical power [7], some caution needs to be taken in interpreting the lack of significant RRs for adverse outcomes in the Omicron era, during which we had fewer participants than in the pre-Omicron era. Pregnant women with SARS-CoV-2 infection appeared to still have a significantly increased RR of preterm birth < 34 weeks during the Omicron period. This increase in preterm birth < 34 weeks during the Omicron period was based on few events, and in the context of multiple comparisons and cross-country heterogeneity, chance findings cannot be ruled out. For the less common outcome of preterm birth < 32 weeks, point estimates remained higher but were no longer statistically significant. It is reassuring that Omicron-era RR point estimates for most outcomes, including preterm birth < 37 weeks, were not increased.

Further limitations relate to timing of infection and outcomes. Although we incorporated analytic safeguards to ensure the correct temporal order of infection occurring before each outcome under investigation, some misclassification could have occurred. For example, we used the midpoint between the last negative and first positive serologic test as the best estimate of infection date for infected women identified through seroconversion. The true infection date could have been later, potentially after the outcome of interest. Fortunately, those identified through seroconversion only represented 2.5% of all infections. Additionally, some outcomes such as preeclampsia were diagnosed during a follow-up visit but may have started earlier, especially if ANC visits were infrequent. Finally, given that most women were enrolled relatively late in pregnancy and only 15% of SARS-CoV-2 infections were identified in the first trimester, we were limited in our ability to reliably evaluate associations for early pregnancy outcomes such as miscarriage.

Our pre-Omicron era findings align with numerous previous studies demonstrating severe disease and elevated risks of adverse pregnancy-related outcomes associated with SARS-CoV-2 infection during pregnancy earlier in the pandemic [1, 2]. Our Omicron era findings differed from another multi-country study by Villar et al., which observed increased composite risks of severe maternal, perinatal, and neonatal morbidity, especially for women with symptomatic COVID-19 [4]. A key methodological distinction lies in the timing and classification of Omicron exposure. The prior study included women who delivered within the first six months of the Omicron era using a global date cutoff. In contrast, our analysis defined Omicron exposure based on country-specific data, including only pregnancies that started when more than 75% of circulating variants were Omicron [10]. Neither ours nor the Villar study conducted genotyping for individual infections, and both approaches likely involved some misclassification with respect to the variant circulating at the time of a given infection. Nonetheless, our study used a stricter definition of Omicron-era pregnancies using the pregnancy start date. Given the rapid and dynamic transition between epidemic waves during the study, we erred on the side of reducing the number of pre-Omicron infections that were misclassified within the “Omicron” group, so that our findings were more representative of the currently relevant period. Additionally, Villar’s study included more HIC data, whereas ours primarily included data from LMICs. A recent study in Botswana found no differences in adverse maternal and neonatal outcomes among women with and without SARS-CoV-2 infection in pregnancy in the period of Omicron predominance [5].

Another potential explanation for differences between our findings and those of Villar et al. may relate to our use of serologic testing to help define those without infection during pregnancy. As part of our sensitivity analyses, we included those with “possible” infection during pregnancy (whose only evidence of infection was a positive serologic test) as part of the uninfected group, which more closely aligns with previous studies that have not incorporated serologic testing. However, this did not result in significantly increased RR estimates in the Omicron era for most outcomes, except for congenital anomalies, which were more likely following infection in the first trimester – a biologically plausible association that should be further explored. The varied findings in our sensitivity analyses likely reflect the effects of both increased power due to larger numbers and increased misclassification (as the seropositive were a mix of those infected during pregnancy and those infected earlier). Differences in specific RR estimates in prior studies, such as findings of an increased risk of stillbirth or maternal death in some studies but not others, may depend on the degree of exposure status misclassification [1, 2].

The decline in risk of adverse outcomes during the Omicron period could be attributed to a decrease in virulence of Omicron variants, or to increased prior immunity, from previous infection, vaccination, or both [13]. Several studies have demonstrated a decrease in overall severity of illness in pregnancy during the Omicron period compared with earlier periods, as observed in nonpregnant adults [3, 14]. We found that among those with SARS-CoV-2 infection, women had lower odds of moderate or severe illness during the Omicron era compared to the pre-Omicron era, regardless of previous vaccination. In turn, controlling for variant period, the odds of moderate to severe illness were almost 40% lower if women were fully vaccinated with the latest dose in pregnancy compared with no vaccination. This finding is important because, among all those with infection in pregnancy, women with more severe illness appeared to have higher risks of adverse outcomes such as maternal near-miss at delivery and emergency caesarean delivery. Although this secondary analysis should be interpreted with caution given evaluation of multiple comparisons with smaller numbers and lack of power to evaluate separately by variant period) [7] it provides some insight into possible mechanisms of reduced risks of adverse pregnancy outcomes as the pandemic has progressed.

Our findings have implications for risk-benefit assessments for interventions in pregnancy, including vaccination. As of January 2026, WHO recommendations were for pregnant women to receive one dose of COVID-19 vaccine in each pregnancy [15], drawing on earlier evidence showing increased risk of adverse outcomes among pregnant women with SARS-CoV-2 infection [1, 2], along with numerous studies showing that vaccines are safe and effective in preventing severe COVID-19 illness for pregnant women and infants, even in the Omicron era [16–19]. The findings of this study contribute to ongoing risk-benefit considerations by showing that the relative risk of adverse outcomes has decreased as the pandemic has progressed. However, potential signals of increased risk of early preterm births for women infected during Omicron-era, and possibly for additional outcomes in sensitivity analyses with greater sample size, suggests that some risks may persist. We did not have data from more recent Omicron sub-variant periods. Continued monitoring can evaluate evolving risk profiles of new variants [20]. The large proportion of women with prior hybrid immunity is reassuring and it may help mitigate a return to the levels of morbidity seen earlier in the pandemic [21]. However, an important consideration is that the lower risks observed in the Omicron period might be related to increased overall vaccination levels—and ongoing vaccination in pregnancy—which we were not fully able to evaluate. A disproportionate number of vaccinated individuals were excluded from the current analysis, preferentially from the uninfected group, based on our use of negative serologic testing at the end of pregnancy to confirm lack of infection. Serologic testing cannot distinguish natural from vaccine-induced antibodies following receipt of inactivated vaccines, which were used by almost a third of those vaccinated and almost 15% of the entire study sample. The separate COVID-19 vaccine evaluation from this study, conducted among the entire study population of 16,005 participants without limiting to those with known infection status, will provide additional information to the risk-benefit equation.

## Conclusions

In summary, this prospective cohort study of pregnant women across 10 countries—one of the largest evaluating COVID-19 and pregnancy, primarily in LMICs—found that SARS-CoV-2 infection during pregnancy was associated with an increased risk of several maternal, perinatal, and neonatal outcomes, particularly during the pre-Omicron era. These findings provide reassurance that, in the Omicron era—when a large proportion of pregnant women have immunity from prior infection and/or vaccination—relative risks appear to be attenuated. However, some evidence of residual increased risk remains, for example for preterm birth before 34 weeks of gestation. As most prior data on COVID-19 and pregnancy outcomes have originated from HICs, this study also highlights the importance of generating high-quality outbreak-related data in LMICs to help safeguard the health of pregnant women and their neonates globally.

## Supplementary Information

Below is the link to the electronic supplementary material.

Supplementary Material 1: Table S1- Table S14. Fig S1- Fig S4

## Data Availability

In compliance with WHO – Ethics Review Committee approval, sharing of individual participant level data is made following the CIOMs guidelines (2016 International ethical guidelines for health-related research involving humans - CIOMS), which indicates in Guideline 12, the need for broad consent and governance structure for sharing of research data. Research datasets, data dictionaries, and analytic codes that support the findings of this study are archived in the access-controlled WHO/HRP e-ARCHIVE repository. In compliance with the WHO Policy on data sharing (Sharing and reuse of health-related data for research purposes: WHO policy and implementation guidance: https://www.who.int/publications/i/item/9789240044968), they may be requested from the corresponding author, Dr. Edna Kara (karae@who.int), by providing a concept note explaining how the data will be used. Anonymized individual participant level research study data, collected through the informed consent, will be made available upon approval of the multi-country editorial board, a governance structure comprising principal investigators and WHO that oversees data sharing, with data share agreement from the country’s principal investigators.

## Abbreviations

Ag: antigen
CI: confidence interval
COVID-19: coronavirus disease 2019
ELISA: enzyme-linked immunosorbent assay
GISAID: Global Initiative on Sharing All Influenza Data
GLMM: generalized linear mixed model
HICs: high-income countries
LMICs: low- and middle-income countries
LMP: last menstrual period
MMMI: modified maternal morbidity and mortality index
N protein: nucleocapsid protein
NICU: neonatal intensive care unit
OR: odds ratio
RR: relative risk
RT-PCR: reverse transcription polymerase chain reaction
S protein: spike protein
SARS-CoV-2: severe acute respiratory syndrome coronavirus 2
SPMMI: severe perinatal morbidity and mortality index
STROBE: Strengthening the Reporting of Observational Studies in Epidemiology
VOC: variant of concern
WHO: World Health Organization
