# Supplementary material for: Maternal, perinatal, neonatal, and postpartum outcomes following Severe Acute Respiratory Syndrome - Coronavirus-2 infection in pregnancy: a World Health Organization multi-country prospective cohort study
Source: BMC Glob Public Health. 2026 Aug 10;4:77. doi: 10.1186/s44263-026-00292-9 (PMC13495363; doi:10.1186/s44263-026-00292-9)
Supplement: Supplementary file 1 [file 44263_2026_292_MOESM1_ESM.docx]

**Supplementary Material 1**

**Table S1**. Study sites in the participating countries, sample size estimates and number of women enrolled

| **Country and study sites** | **Sample size** | **Enrolment** | **Remarks** |
| --- | --- | --- | --- |
|  | **Actual / Target** | **Started / Ended** |  |
| **Argentina:** two level III maternity hospitals (one private and one public). Referral hospitals in the capital cities of two Argentina regions. | 283 / 1,680 | Jan 2022 / Apr 2023 | Inclusion during pregnancy and postpartum (1.1% in 2nd trimester, 61.5% in 3rd, 37.5% postpartum). Women were recruited in hospitals. |
| **Brazil:** Nine maternity hospitals (Six public, one private, and two mixed). Four Brazilian regions. | 3,109 / 2,902 | Aug 2021 / Dec 2022 | Inclusion during pregnancy and postpartum (11% in 1st trimester, 25.5% in 2nd, 27.0% in 3^rd^, 36.5% postpartum). |
| **Burkina Faso**: Ten public health facilities. One Burkina Faso region. | 1,785 / 1,652 | Feb 2022 / Nov 2022 | Only inclusion during pregnancy: complete by trimester. |
| **Chile:** Two Maternity hospitals. Two Chilean regions. | 288 / 2,318 | Mar 2023 / Oct 2023 | Only inclusion during postpartum. |
| **Ghana:** Five public health facilities. One Ghanaian region. | 1,444 / 1,435 | Jun 2021 / Oct 2021 | Only inclusion during pregnancy: 1% in 1st trimester, 16% in 2nd, 83% in 3rd trimester. |
| **Kenya:** Two referral hospitals in the capital city (One public and one private). One Kenyan region. | 1,717 / 1,710 | Jun 2021 / Nov 2022 | Around 95% inclusion during pregnancy (8% 1st trimester, 23.7% in 2nd, 68.3% in 3rd trimester). Women were recruited in hospitals. |
| **Pakistan:** Six health facilities (Four public and two private). Five Pakistan regions. | 5,398 / 5,236 | Jun 2021 / Oct 2022 | Only inclusion during pregnancy: 10.9% in 1st trimester, 42.7% in 2nd, and 46.4% in 3rd trimester. |
| **Philippines:** Five public hospitals. National Capital Region. | 733 / 820 | Sep 2021 / Sep 2022 | Inclusion mostly in the late 3rd trimester. Around inclusion during pregnancy (0.41% in 1st trimester, 0% in 2^nd^, 99.59 % in 3rd trimester). |
| **Spain:** One public maternity hospital | 53 / 120 | Jan 2021 / Feb 2022 | — |
| **Tunisia:** One referral public maternity hospital | 1,195 / 877 | Jul 2021 / Dec 2022 | Most included in 3rd trimester. |
| **Total 10 countries and 43 health facilities** | **16,007 / 18,750** | **Jan 2021** / **Oct 2023** | — |

The pooled analysis includes data from all 10 countries that implemented the core WHO protocol [1](<https://www.who.int/publications/i/item/WHO-2019-nCoV-pregnancy-and-neonates-2022.1>). Target sample sizes varied, as each country calculated an individual country-specific target sample size to evaluate whether SARS-CoV-2 infection during pregnancy increases the risk of adverse pregnancy outcomes, considering national epidemic contexts at the time of study initiation, key outcomes of interest locally, and baseline estimates of the adverse outcomes in the relevant settings.

In 2021, after COVID-19 vaccines became available, WHO amended the core protocol to include assessment of vaccine use. In addition, updated sample size calculations were done to account not just for vaccination objectives, but also for the increasing proportion of women expected to have previous exposure to SARS-CoV-2 infection as the pandemic progressed. This increased the proportion with existing antibodies at baseline, which thus increased uncertainty about who was infected during pregnancy. For this reason, several countries continued to enroll participants beyond their original sample size targets.

The final updated version of the protocol, with the sample size considerations for the pooled analysis, was published online in 2022 (<https://www.who.int/publications/i/item/WHO-2019-nCoV-pregnancy-and-neonates-2022.1>). With 80% power, 5% Type 1 error, and baseline incidence of 10%, sample sizes for detecting a 25% increased risk in preterm birth in the infected group relative to the uninfected group are 2504 participants with infection and 2504 without.

Delays related to local ethical approvals and import and availability of study test kits substantially impacted recruitment in Chile and Argentina, leading to premature closure before reaching the target sample sizes [2].

References

1. Generic protocol: a prospective cohort study investigating maternal, pregnancy and neonatal outcomes for women and neonates infected with SARS-CoV-2. 1 Nov 2022. [Accessed 2026 Mar 5]
2. Costa ML, Souza RT, Cecatti JG, Gottlieb SL, Delnord M, Thwin SS, et al. Implementation of the multicountry WHO COVID-19 pregnancy cohort study: challenges and lessons learned during the pandemic. Reprod Health. 2025 Nov 12;22(1):231. doi:10.1186/s12978-025-02080-4.

**Table S2**. Detailed definitions of outcome variables and associated denominators for analysis

| **Outcome** | **Definition** | **Denominator^¥^** |
| --- | --- | --- |
| **A. Maternal and pregnancy outcomes** | | |
| **Miscarriage** | Includes both spontaneous - a condition caused by immunological factors, abnormal ovum or uterine body, maternal disease or infection, or cervical incompetence. This condition is characterised by non-induced embryonic or fetal death or passage of products of conception prior to 22 weeks (or 28 weeks) gestation or weighing less than 500 grams and missed abortion - a condition caused by genetic abnormality, abnormal cell division, or poor quality ovum or sperm. This condition is characterised by a failed pregnancy, immature fetal or embryonic death that is not expelled from the uterus for at least 8 weeks, and diminished uterine size. This condition may also present with maternal infection, blood clotting, fetal calcification, and resorption of conception products. Confirmation is by imaging.) [1] | All completed pregnancies enrolled at < 20 weeks gestation |
| **Maternal haemorrhage** | Antepartum haemorrhage is defined as bleeding from or into the genital tract, occurring from 24+0 weeks of pregnancy and prior to the birth of the baby [2]. This outcome also includes intrapartum haemorrhage and postpartum haemorrhage observed immediately following labour and delivery. | All completed pregnancies |
| **Preeclampsia or eclampsia** | Preeclampsia: de-novo development of concurrent hypertension and proteinuria  Eclampsia: generalized seizures in women with pre-eclampsia not due to another cause, e.g., epilepsy | All completed pregnancies |
| **Hypertensive disorders of pregnancy (including preeclampsia, eclampsia)** | Any diagnosis of hypertensive disorders of pregnancy during the current pregnancy, including preeclampsia, eclampsia, and hemolysis, elevated liver enzyme levels, and low platelet levels (HELLP) syndrome. | All completed pregnancies |
| **Thromboembolic disease** | Either venous thrombosis or embolic event during the current pregnancy. | All completed pregnancies |
| **Preterm labour** | Onset of labour prior to 37 weeks gestation. | All completed pregnancies |
| **Placental abruption** | Clinical diagnosis of placental abruption. | All completed pregnancies |
| **Maternal near-miss indicators at delivery** | Woman who nearly died but survived a complication that occurred at the time of delivery. At least one of the following indicators: acute cyanosis, Oxygen saturation <90% for > 60 minutes, use of continuous vasoactive drugs, gasping, PaO2/FiO2 <200 mmHg, hysterectomy following infection or haemorrhage, Respiratory rate >40/min or <6/min, Creatinine ≥ 300 micromol/l or ≥ 2.5 mg/dl, Transfusion ≥ 5 units red cells, shock, Bilirubin > 100 micromol/l or > 6.0 mg/dl, Intubation and ventilation for ≥ 60 minutes not related to anesthesia, Oliguria non-responsive to fluids /diuretics, pH <7.1, dialysis, clotting failure, lactate >5, cardiopulmonary resuscitation (CPR), Loss of consciousness lasting ≥ 12 hours, Acute thrombocytopenia (<50,000 platelets), Loss of consciousness and absence of pulse/heartbeat, Loss of consciousness and the presence of glucose and ketoacids in urine, stroke, uncontrollable fit/total paralysis, jaundice in the presence of preeclampsia. | All completed pregnancies |
| **Delivery mode** | Birth of one or more neonates from the uterus either spontaneously, assisted, or by caesarean section [3]. | All completed pregnancies ending in a birth (including all livebirths AND stillbirths at or after 22 weeks gestation) |
| **Maternal death** | Death from any cause related to or aggravated by pregnancy or its management (excluding accidental or incidental causes) during pregnancy and childbirth or within 42 days of termination of pregnancy, irrespective of the duration and site of the pregnancy.  Maternal causes of death may be subdivided into two  groups: (1) direct obstetric deaths, resulting from obstetric complications of the pregnant state (pregnancy, labour and the puerperium), from interventions, omissions, incorrect treatment or from a chain of events resulting from any of the above; and (2) indirect obstetric deaths, resulting from  previous existing disease or disease that developed during pregnancy and that  was not due to  direct obstetric causes, but was aggravated by the physiological effects of pregnancy [4]. | All completed pregnancies |
| **Modified maternal morbidity and mortality index (MMMI)** | Includes at least 1 of the following pregnancy-related morbidities: haemorrhage at the time of labour and delivery; hypertensive disorders of pregnancy (including preeclampsia/eclampsia); thromboembolic disease in pregnancy; preterm labour; placental abruption; OR near-miss indicator at delivery; OR death (adapted from Villar et al., 2021 [5]) | All completed pregnancies |
| **B. Perinatal & Neonatal Outcomes** | | |
| **Stillbirth** | Fetal death occurring before birth after a selected, predefined duration of gestation, recorded in the patient records. Both intrapartum stillbirth and antepartum stillbirth are considered. The predefined duration of gestation varies between 22 and 28 weeks across sites. Antepartum stillbirth: Fetal death occurs prior to the evidence of labour. Intrapartum stillbirth: Fetal death occurs during labour and before delivery [6] [7] | All births ending in livebirth or in fetal death at or after 22 (or 28) weeks of gestation |
| **Preterm birth** | Preterm is defined as babies born alive before 37 weeks of pregnancy are completed. Additional sub-categories of preterm birth evaluated, based on gestational age: less than 32 weeks (includes “extremely” and “very” preterm birth), and less than 34 weeks (also includes “moderate” preterm birth) [8] | Main analysis: Infected: positive SARS-CoV-2 test <37 weeks; Uninfected: all livebirths    Additional sample restrictions for sensitivity analyses:    1; Infected: positive SARS-CoV-2 test <37 weeks, enrolled <37 weeks; Uninfected: enrolled <37 weeks  2. Infected: positive SARS-CoV-2 test <37 weeks; Uninfected: negative SARS-CoV-2 test <37 weeks |
| **Low birth weight** | First weight of <2,500 grams recorded in the birth register or patient record  First weight <1,500 grams recorded in the birth register or patient record [9] | All livebirths |
| **Any congenital anomaly** | Any clinical diagnosis of congenital anomaly  Neural tube defects, microcephaly, malformation of the ear (microtia/anotia), heart defects, orofacial clefts, malformation of digestive system, malformation of genital organs, abdominal wall defects, chromosomal abnormalities, reduction defect of limb, talipes equinovarus/clubfoot.  Or other congenital anomaly as determined by the study site | All births ending in livebirth or in fetal death at or after 22 (or 28) weeks of gestation |
| **Perinatal death** | Fetal death before or during birth at or after 28 weeks gestation + early neonatal deaths (within the first 7 days of life) [10]. The predefined duration of gestation varies between 22 and 28 weeks across sites. | All births ending in livebirth or in fetal death at or after 22 (or 28) weeks gestation |
| **Neonatal mortality** | Neonatal deaths (deaths among live births during the first 28 completed days of life) may be subdivided into early neonatal deaths, occurring during the first 7 days of life, and late neonatal deaths, occurring after the 7th day but before the 28th completed day of life [11]. | All livebirths |
| **Neonatal admission to NICU** | Admission to a Neonatal Intensive Care Unit (or the equivalent) at birth. | All livebirths |
| **Severe perinatal morbidity and mortality index (SPMMI)** | Includes fetal death, at least 1 of the severe neonatal conditions:  hypoxic-ischemic encephalopathy or sepsis, OR admission to the neonatal ICU (NICU) for 7 days, or longer, OR neonatal death before hospital discharge (index adapted from Villar et al, 2021 [5]) | All births ending in livebirth or in fetal death at or after 22 weeks gestation |
| **C. Postpartum outcomes** | | |
| **Maternal postpartum haemorrhage** | Clinical diagnosis up to 42 days [2]. Excludes haemorrhage intrapartum and observed immediately following labour and delivery. | All completed pregnancies followed up for 42 days |
| **Maternal infection or endometritis postpartum** | Clinical diagnosis of maternal infection of any kind or infection of the inner lining of the uterus. | All completed pregnancies followed up for 42 days |
| **Maternal rehospitalization** | Any hospitalization/re-admission postpartum (between discharge & 42 weeks) | All completed pregnancies followed up for 42 days |

^¥^ All analyses incorporate temporal restrictions whereby outcomes occurring prior to COVID-19 test date are recoded to ensure temporal plausibility of the exposure-outcome relationship.

**REFERENCES**

1. World Health Organization. ICD‑11 for Mortality and Morbidity Statistics. JA03 Missed abortion; JA00.0 Spontaneous abortion. Available from: <https://icd.who.int/browse11/l-m/en#/http%3a%2f%2fid.who.int%2ficd%2fentity%2f721991699>; <https://icd.who.int/browse11/l-m/en#/http%3a%2f%2fid.who.int%2ficd%2fentity%2f696502028> [Accessed 2026 Mar 5]
2. World Health Organization. WHO recommendations for the prevention and treatment of postpartum haemorrhage. Geneva: WHO; 2012. Available from: [https://www.who.int/publications/i/item/9789241548502](https://www.who.int/publications/i/item/9789241548502?utm_source=chatgpt.com) [Accessed 2026 Mar 5]
3. World Health Organization. ICD‑11 for Mortality and Morbidity Statistics. Delivery. Available from: <https://icd.who.int/browse11/l-m/en#/http%3a%2f%2fid.who.int%2ficd%2fentity%2f973282267> [Accessed 2026 Mar 5]
4. World Health Organization Global Health Observatory. Maternal mortality ratio (per 100 000 live births); Haemorrhage – number of maternal cases. Available from: <https://www.who.int/data/gho/data/indicators/indicator-details/GHO/maternal-mortality-ratio-(per-100-000-live-births)>; <https://www.who.int/data/gho/indicator-metadata-registry/imr-details/3569> [Accessed 2026 Mar 5]
5. Villar J, Ariff S, Gunier RB, Thiruvengadam R, Rauch S, Kholin A, et al. Maternal and neonatal morbidity and mortality among pregnant women with and without COVID‑19 infection: the INTERCOVID multinational cohort study. JAMA Pediatr. 2021;175(8):817–26. doi:10.1001/jamapediatrics.2021.1050
6. Stuurman AL, Riera M, Lamprianou S, Perez‑Vilar S, Anderson SA, Mangtani P, et al. Vaccine safety surveillance in pregnancy in low‑ and middle‑income countries using GAIA case definitions: a feasibility assessment. Vaccine. 2018;36(45):6736–43. doi:10.1016/j.vaccine.2018.09.033
7. Tavares Da Silva F, Gonik B, McMillan M, Keech C, Dellicour S, Bhange S, et al; Brighton Collaboration Stillbirth Working Group. Stillbirth: Case definition and guidelines for data collection, analysis, and presentation of maternal immunization safety data. Vaccine. 2016;34(49):6057–68. doi:10.1016/j.vaccine.2016.03.044
8. World Health Organization. Preterm birth. 2023. Available from: <https://www.who.int/news-room/fact-sheets/detail/preterm-birth> [Accessed 2026 Mar 5]
9. World Health Organization. Low birthweight. Available from: <https://www.who.int/data/nutrition/nlis/info/low-birth-weight> [Accessed 2026 Mar 5]
10. Barfield WD; Committee on Fetus and Newborn. Standard terminology for fetal, infant, and perinatal deaths. Pediatrics. 2016;137(5):e20160551. doi:10.1542/peds.2016-0551
11. United Nations Economic Commission for Europe. Indicator 3.2.2: Neonatal mortality rate, per 1,000 live births. Available from: <https://w3.unece.org/SDG/en/Indicator?id=92> [Accessed 2026 Mar 5]

**Table S3.** Country-specific dates of variant of concern (VOC) predominance (≥75% of sequenced SARS-CoV-2 cases), based on GISAID data

| **Country** | **Pre-Omicron variants (≥75% period)** | **Omicron start date** |
| --- | --- | --- |
| Argentina | Delta: 22 Oct – 12 Dec 2021 | 30 Dec 2021 |
| Brazil | Gamma: 1 Mar – 26 Jul 2021; Delta: 28 Aug – 16 Dec 2021 | 28 Dec 2021 |
| Burkina Faso | Delta: 27 Sep – 20 Dec 2021 | 5 Jan 2022 |
| Chile | Delta: 15 Sep – 12 Dec 2021 | 27 Dec 2021 |
| Ghana | Delta: 6 Jul – 16 Nov 2021 | 30 Nov 2021 |
| Kenya | Delta: 17 Jun – 19 Nov 2021 | 4 Dec 2021 |
| Pakistan | Delta: 14 Jul – 22 Nov 2021 | 10 Jan 2022 |
| Philippines | Delta: 1 Aug – 15 Oct 2021 | 16 Nov 2021 |
| Spain | Alpha: 23 Mar – 25 May 2021; Delta: 12 Jul – 10 Dec 2021 | 2 Jan 2022 |
| Tunisia | Delta: 31 Aug – 19 Dec 2021 | 7 Jan 2022 |

*Note: Dates indicate the period during which each variant constituted ≥75% of sequenced SARS-CoV-2 cases in country-level GISAID data. Pregnancies starting before the country-specific Omicron start date were classified as pre-Omicron; those starting on or after were classified as Omicron.*

**Table S4.** SARS-CoV-2 infection status categories by country

| **Country** | **Confirmed infected** | **Probably infected** | **Possibly infected** | **Probably uninfected** | **Confirmed uninfected** | **Uncertain / unknown** | **Cases by country** |
| --- | --- | --- | --- | --- | --- | --- | --- |
| Argentina | 134  (6%) | 0  (0%) | 4  (0.1%) | 9  (2%) | 1  (0.1%) | 135  (3%) | 283  (1.8%) |
| Brazil | 588  (27%) | 8  (7%) | 481  (7%) | 116  (29%) | 270  (25.6%) | 1,646 (34%) | 3,109  (19.4%) |
| Burkina Faso | 35  (2%) | 14  (12%) | 1,618 (22%) | 6  (2%) | 2  (0.2%) | 110  (2%) | 1,785  (11.2%) |
| Chile | 43  (2%) | 0  (0%) | 26  (0.4%) | 5  (1%) | 15  (1.4%) | 199  (4%) | 288  (1.8%) |
| Ghana | 94  (4%) | 0  (0%) | 742  (10%) | 11  (3%) | 376  (36%) | 221  (5%) | 1,444  (9%) |
| Kenya | 230  (11%) | 20  (17%) | 1,119 (15%) | 33  (8%) | 126  (12%) | 189  (4%) | 1,717  (10.7%) |
| Pakistan | 362  (17%) | 66  (57%) | 2,682  (37%) | 100  (25%) | 232  (22%) | 1,956 (40%) | 5,398  (33.7%) |
| Philippines | 394  (18%) | 1  (1%) | 205  (3%) | 1  (0.2%) | 1  (0.1%) | 131  (3%) | 733  (4.6%) |
| Spain | 16  (1%) | 0  (0%) | 0  (0%) | 4  (1%) | 1  (0.1%) | 32  (1%) | 53  (0.3%) |
| Tunisia | 291  (13%) | 7  (6%) | 455  (6%) | 117  (29%) | 29  (3%) | 296  (6%) | 1,195  (7.5%) |
| Total | 2,187 | 116 | 7,332 | 402 | 1,053 | 4,915 | 16,005 |

 **Fig S1.** Number of pregnancies by their start date and year/quarter

**Note:** Figure includes 15,912 pregnancies. 93 pregnancies with missing pregnancy start date could not be classified by quarter and are excluded from this figure (total study population N = 16,005).

**Fig S2.** Number of pregnancies by variant of concern (VOC) at 75% predominance in the country

**Table S5.** Medical conditions of pregnant women by SARS-CoV-2 infection status

| **Characteristic** | **Confirmed or Probably Infected**  N = 2,303*^1^* | **Confirmed or Probably Uninfected**  N = 1,455*^1^* | **Unadjusted p-value***^2^* |
| --- | --- | --- | --- |
| **Heart disease prior to and during current pregnancy** | | | 0.196 |
| Yes | 34 (1.5%) | 14 (1.0%) |  |
| No | 2,193 (98.5%) | 1,425 (99.0%) |  |
| Missing /Unknown (M/U) | 76 | 16 |  |
| **Lung disease prior to and during current pregnancy** | | | 0.086 |
| Yes | 85 (3.8%) | 39 (2.7%) |  |
| No | 2,144 (96.2%) | 1,402 (97.3%) |  |
| M/U | 74 | 14 |  |
| **Kidney or Liver disease** | | | 0.298 |
| Yes | 37 (1.7%) | 17 (1.2%) |  |
| No | 2,189 (98.3%) | 1,423 (98.8%) |  |
| Missing/Unknown | 77 | 15 |  |
| **Hypertension prior to and during the current pregnancy** | | | 0.642 |
| Yes | 162 (7.3%) | 98 (6.8%) |  |
| No | 2,069 (92.7%) | 1,343 (93.2%) |  |
| M/U | 72 | 14 |  |
| **Preexisting Type 1 or Type 2 diabetes prior to and during current pregnancy** | | | 0.293 |
| Yes | 89 (4.0%) | 47 (3.3%) |  |
| No | 2,140 (96.0%) | 1,393 (96.7%) |  |
| M/U | 74 | 15 |  |
| **HIV prior to and during the current pregnancy** | | | 0.952 |
| Yes | 40 (1.8%) | 27 (1.9%) |  |
| No | 2,172 (98.2%) | 1,399 (98.1%) |  |
| M/U | 91 | 29 |  |
| **Tuberculosis prior to and during the current pregnancy** | | | 0.334 |
| Yes | 6 (0.3%) | 1 (0.1%) |  |
| No | 2,184 (99.7%) | 1,414 (99.9%) |  |
| M/U | 113 | 40 |  |
| **Body Mass Index (BMI) >= 30 kg/m²** | | | 0.513 |
| Yes | 306 (25.5%) | 268 (26.8%) |  |
| No | 895 (74.5%) | 732 (73.2%) |  |
| M/U | 1,102 | 455 |  |
| **BMI < 18.5 kg/m²** | | | 0.621 |
| Yes | 34 (2.8%) | 24 (2.4%) |  |
| No | 1,169 (97.2%) | 976 (97.6%) |  |
| M/U | 1,102 | 455 |  |
| *^1^* n (%) | | | |
| *^2^* Pearson’s Chi-squared test; Fisher’s exact test | | | |

**Table S6.** Selected participant characteristics by SARS-CoV-2 infection status during pregnancy

| **Characteristic** | **Confirmed or probably infected**  N = 2,303*^1^* | | **Confirmed or probably uninfected**  N = 1,455*^1^* | | **Possibly infected**  N = 7,332 | **Uncertain / Unknown infection status**  N = 4,915 |  |  |
| --- | --- | --- | --- | --- | --- | --- | --- | --- |
| **Maternal age at enrollment** | | | | |  |  |  |  |
| **In years^1^:** | 30 (26, 35) | | 31 (27, 35) | | 28 (24,33) | 29 (25,34) |  |  |
| **In years by groups:** | | | | |  |  |  |  |
| <20 | 67 (2.9) | | 36 (2.5) | | 467 (6.4) | 170 (3.5) |  |  |
| 20-35 | 1,712 (74.95.0) | | 1,060 (73.2) | | 5,774 (78.9) | 3,839 (79.3) |  |  |
| >=36 | 5075 (22.1) | | 352 (24.3) | | 1,073 (14.7) | 834 (17.2) |  |  |
| Missing/Unknown | 19 | | 7 | | 18 | 72 |  |  |
| **Gestational age at enrollment** | | | | |  |  |  |  |
| **In weeks^1^:** | 36 (26, 39) | | 36 (30, 38) | | 27 (20,36) | 27 (18,37) |  |  |
| **In weeks by group:** |  | |  | |  |  |  |  |
| <14 weeks | 147 (6.4) | | 63 (4.3) | | 706 (9.6) | 610 (12.6) |  |  |
| 14-27 weeks | 505 (22.0) | | 155 (10.7) | | 3,057 (41.7) | 1,913 (39.7) |  |  |
| > 27 weeks | 1,6464 (71.6) | | 1,235 (85.0) | | 3,561 (48.6) | 2,300 (47.7) |  |  |
| Missing/Unknown | 7 | | 2 | | 8 | 92 |  |  |
| **Enrolled in the 48 hours after delivery** | | | | |  |  |  | |
| No | 2,0586 (89.3) | | 1,265 (86.9) | | 7,033 (95.9) | 4,384 (89.2) |  | |
| Yes | 247 (10.7) | | 190 (13.1) | | 299 (4.1) | 531 (10.8) |  | |
| **Type of facility** | | | | |  |  |  | |
| Primary | 77 (3.5) | | 167 (12.1) | | 1,818 (26.5) | 198 (4.4) |  | |
| Secondary | 41 (1.8) | | 105 (7.6) | | 345 (5.0) | 198 (4.4) |  | |
| Tertiary | 2,107 (94.7) | | 1,113 (80.4) | | 4,688 (68.4) | 4,092 (91.2) |  | |
| Missing/Unknown | 78 | | 70 | | 481 | 427 |  | |
| **Number of pregnancies (including current)** | | | | |  |  |  |  |
| Primigravida | 607 (26.5) | | 449 (31.0) | | 2,160 (29.5) | 1,553 (32.0) |  |  |
| Multigravida | 1,680 (73.5) | | 1,000 (69.0) | | 5,165 (70.5) | 3,300 (68.0) |  |  |
| Missing/Unknown | 16 | | 6 | | 7 | 62 |  |  |
| **Marital status** | | | | |  |  |  |  |
| Single or Previously Married | 553 (24.3) | | 195 (13.5) | | 677 (9.3) | 774 (16.1) |  |  |
| Married/Cohabiting | 1,720 (75.7) | | 1,248 (86.5) | | 6,632 (90.7) | 4,035 (83.9) |  |  |
| Missing/Unknown | 30 | | 12 | | 23 | 106 |  |  |
| **Highest level of education completed** | | | | |  |  |  |  |
| Basic Education | 472 (21.5) | | 416 (29.0) | | 3,316 (45.7) | 1,486 (31.1) |  |  |
| Secondary Education | 809 (36.8) | | 493 (34.4) | | 2,303 (31.8) | 1,844 (38.6) |  |  |
| Higher Education | 919 (41.8) | | 524 (36.6) | | 1,630 (22.5) | 1,449 (30.3) |  |  |
| Missing/Unknown | 103 | | 22 | | 83 | 136 |  |  |
| **Occupation** |  | |  | |  |  |  |  |
| Employed or Self-employed | 964 (41.8) | | 789 (54.2) | | 2,192 (29.9) | 1,660 (33.8) |  |  |
| Unemployed or Informal or Other | 1,325 (57.5) | | 660 (45.4) | | 5,133 (70.0) | 3,194 (65.0) |  |  |
| Missing/Unknown | 15 | | 6 | | 7 | 61 |  |  |
| **Alcohol consumption within the past 30 days** | | | | |  |  |  |  |
| Yes | | 50 (2.2) | 19 (1.3) | | 162 (2.2) | 127 (2.6) |  |  |
| No | | 2,209 (97.8) | 1,423 (98.7) | | 7,155 (97.8) | 4,702 (97.4) |  |  |
| Missing/Unknown | | 44 | 13 | | 15 | 86 |  |  |
| **Currently smoke any tobacco products** | | | | |  |  |  |  |
| Yes | | 41 (4.0) | 24 (1.6) | | 27 (0.6) | 90 (1.8) |  |  |
| No | | 2,211 (96.0) | 1,421 (98.4) | | 7,285 (99.4) | 4,731 (96.3) |  |  |
| Missing/Unknown | | 0 | 0 | | 27 | 90 |  |  |
| **Presence of medical conditions^§^** | | | | |  |  |  | |
| 0 | | 1,604 (70.5) | | 993 (68.5) | 5,788 (79.1) | 3,235 (66.8) |  | |
| 1 | | 475 (20.9) | | 341 (23.5) | 1,064 (14.5) | 1,058 (21.8) |  | |
| 2 or more | | 142 (6.2) | | 92 (6.3) | 147 (2.0) | 379 (7.8) |  | |
| Missing/Unknown | 27 | | 6 | | 15 | 69 |  |  |
| **Vaccination status*** | | | | |  |  |  |  |
| Never | 753 (35.5) | | 630 (45.5) | | 5,351 (74.9) | 586 (12.8) |  |  |
| Partially | 169 (8.0) | | 162 (11.7) | | 535 (7.5) | 710 (15.5) |  |  |
| Fully, last dose before pregnancy | 502 (23.7) | | 201 (14.5) | | 577 (8.1) | 1,546 (33.8) |  |  |
| Fully, last dose during pregnancy | 697 (32.9) | | 393 (28.4) | | 678 (9.5) | 1,731 (37.8) |  |  |
| Missing/Unknown | 182 | | 69 | | 191 | 342 |  |  |
| *^1^* Median (IQR); n (%) | | | | | | | |  |
| *^2^* Pearson’s Chi-squared test; Wilcoxon rank sum test | | | | | | | |  |

*Vaccination status represents the status at the end of follow-up, rather than baseline

**Fig S3.** Association between SARS-CoV-2 exposure and maternal, pregnancy, perinatal, neonatal, and postpartum outcomes.

Results are presented for the overall population (gray), the population restricted to pre-Omicron cases (coral), and the population restricted to Omicron cases (teal). Dots indicate point estimates, and horizontal lines represent 95% confidence intervals. Models were adjusted for maternal age and the number of underlying conditions.

**Table S7**. Sensitivity analysis with restricted samples for preterm birth outcomes

|  |  | **Adjusted random-effects model** | | |
| --- | --- | --- | --- | --- |
|  |  | **Relative Risk (95% Confidence Interval); p-value^2^** | | |
| **OUTCOME: Preterm birth <37 weeks** | | **Overall sample** | **Pre-Omicron** | **Omicron** |
| **Primary Definition** | All livebirths  Infected: positive SARS-CoV-2 test <37 weeks;  Non-infected: all livebirths | 1.53  (1.26,1.84)  p = < 0.001 | 1.73  (1.31,2.23)  p = 0.0001 | 1.02  (0.68,1.53)  p = 0.9102 |
| **Restricted Sample Definition 1** | Infected: positive SARS-CoV-2 test <37 weeks, enrolled <37 weeks;  Non-infected: enrolled <37 weeks | 1.14  (1.04, 1.25)  p = 0.0063 | 1.23  (1.02, 1.48)  p = 0.0274 | 0.96  (0.77,1.21)  p = 0.7454 |
| **Restricted Sample Definition 2** | Infected: positive SARS-CoV-2 test <37 weeks;  Non-infected: negative SARS-CoV-2 test <37 weeks | 0.98  (0.80,1.20)  p = 0.8256 | 1.03  (0.82, 1.29)  p = 0.8312 | 0.86  (0.61,1.24)  p = 0.850 |
|  |  |  |  |  |
| **OUTCOME: Preterm birth <34 weeks** | | **Overall sample** | **Pre-Omicron** | **Omicron** |
| **Primary Definition** | All livebirths  Infected: positive SARS-CoV-2 test <34 weeks;  Non-infected: all livebirths | 3.01  (1.74,5.21)  p = 0.0001 | 3.67  (1.92,7.04)  p = 0.0001 | 1.81  (1.07,3.05)  p = 0.0272 |
| **Restricted Sample Definition 1** | Infected: positive SARS-CoV-2 test <34 weeks, enrolled <34 weeks;  Non-infected: enrolled <34 weeks | 1.36  (1.10, 1.68)  p = 0.0050 | 1.54  (1.06,2.23)  p = 0.0244 | 1.12  (0.95,1.33)  p = 0.1678 |
| **Restricted Sample Definition 2** | Infected: positive SARS-CoV-2 test <34 weeks;  Non-infected: negative SARS-CoV-2 test <34 weeks | 1.25  (0.85,1.82)  p = 0.2538 | 1.26  (0.73,2.16)  p = 0.4063 | 1.51  (0.92, 2.48)  p = 0.1054 |
|  |  |  |  |  |
| **OUTCOME: Preterm birth < 32 weeks** | | **Overall sample** | **Pre-Omicron** | **Omicron** |
| **Primary Definition** | All livebirths  Infected: positive SARS-CoV-2 test <32 weeks;  Non-infected: all livebirths | 4.56  (1.50,13.81)  p = 0.0074 | 7.62  (2.16,26.85)  p = 0.0016 | 1.62  (0.70,3.72)  p = 0.2584 |
| **Restricted Sample Definition 1** | Infected: positive SARS-CoV-2 test <32 weeks, enrolled <32 weeks;  Non-infected: enrolled <32 weeks | 1.32  (1.01, 1.71)  p = 0.0398 | 2.02  (1.19,3.43)  p = 0.0093 | 0.95  (0.78, 1.17)  p = 0.6501 |
| **Restricted Sample Definition 2** | Infected: positive SARS-CoV-2 test <32 weeks;  Non-infected: negative SARS-CoV-2 test <32 weeks | 1.38  (0.64, 3.01)  p = 0.4137 | 1.89  (0.58, 6.26)  p = 0.2920 | 0.95  (0.68, 1.33)  p = 0.7621 |
|  |  |  |  |  |

**Table S8.** Sensitivity analysis 1 (Possibly infected group added as ‘SARS-CoV-2 infection’): Maternal and pregnancy outcomes by SARS-CoV-2 infection status^¥^

| **Outcome^§^** | **Confirmed or Probably Infected^£^**  N = 9,635*^1^* | **Confirmed or Probably Uninfected^£^**  N = 1,455*^1^* | **Adjusted (Infected vs Uninfected) random-effects model**  **Adjusted Relative Risk (95% Confidence Intervals); p-value^2^** | | |
| --- | --- | --- | --- | --- | --- |
|  |  |  | **Overall sample**  **(Events / Total)** | **Restricted to pre-Omicron**  **Period**  **(Events / Total)** | **Restricted to Omicron**  **Period**  **(Events / Total)** |
| **Miscarriage (before 22 weeks of gestation)** | | | **85/2,027** | **36/,1095** | **49/932** |
| Yes | 80 (4.2) | 5 (4.9) | 1.03  (0.67,1.57)  p = 0.9113 | 1.17 (0.44,3.14)  p = 0.7519 | 1.04 (0.71,1.52)  p = 0.8375 |
| No | 1,849 (95.9) | 97 (95.1) |  |  |  |
| At risk | 1,929 | 102 |  |  |  |
| **Miscarriage (before 28 weeks)** | | | **92/2,036** | **38/1,098** | **54/938** |
| Yes | 88 (4.5) | 5 (4.9) | 1.05  (0.69,1.60)  p = 0.8327 | 1.24 (0.49,3.18)  p = 0.6508 | 1.06 (0.72,1.54)  p = 0.7783 |
| No | 1,851 (95.5) | 97 (95.1) |  |  |  |
| At risk | 1,939 | 102 |  |  |  |
| **Haemorrhage (Antepartum, Intrapartum, or Postpartum)** | | | **248/10,300** | **176/8,007** | **72/2,293** |
| Yes | 213 (2.4) | 36 (2.6) | 0.91  (0.49,1.69)  p = 0.7567 | 1.11  (0.58,2.12)  p = 0.7505 | 0.43  (0.29,0.62)  p < 0.001 |
| No | 8,749 (97.6) | 1,344 (97.4) |  |  |  |
| At risk | 8,962 | 1,380 |  |  |  |
| **Preeclampsia/eclampsia** | | | **544/10,436** | **415/8,063** | **129/2,373** |
| Yes | 461 (5.1) | 84 (6.1) | 1.33  (1.06,1.67)  p = 0.0145 | 1.45 (1.13,1.86)  p = 0.0028 | 0.86 (0.57,1.30)  p = 0.4840 |
| No | 8,631 (94.9) | 1,305 (94.0) |  |  |  |
| At risk | 9.092 | 1,389 |  |  |  |
| **Hypertensive disorders of pregnancy** | | | **711/10,438** | **533/8,065** | **178/2,373** |
| Yes | 604 (6.6) | 109 (7.9) | 1.33  (1.0,1.77)  p = 0.0519 | 1.52  (1.17,1.96)  p = 0.0016 | 0.81  (0.58,1.14)  p = 0.2271 |
| No | 8,490 (92.2) | 1,280 (92.2) |  |  |  |
| At risk | 9.094 | 1,389 |  |  |  |
| **Thromboembolic disease** | | | **12/10,437** | **11/8,064** | **1/2,373** |
| Yes | 9 (0.1) | 3 (0.2) | 0.55  (0.19,1.63)  p = 0.2787 | 0.55  (0.17, 1.76)  p = 0.3145 | — |
| No | 9,082 (99.9) | 1,385 (99.8) |  |  |  |
| At risk | 9,091 | 1,388 |  |  |  |
| **Preterm labour** | | | **589/7,734** | **409/5,679** | **180/2,055** |
| Yes | 530 (7.6) | 60 (7.6) | 0.90  (0.80,1.02)  p = 0.1014 | 0.81  (0.72,0.91)  p = 0.0003 | 1.26  (0.96,1.64)  p = 0.0945 |
| No | 6,434 (92.4) | 731 (92.4) |  |  |  |
| At risk | 6,964 | 791 |  |  |  |
| **Placental abruption** | | | **47/10,433** | **30/8,061** | **17/2,372** |
| Yes | 40 (0.4) | 7 (0.5) | 0.88 (0.48,1.59)  p = 0.6644 | 0.69 (0.38,1.25)  p = 0.2175 | 1.17 (0.43,3.21)  p = 0.7585 |
| No | 9,047 (99.6) | 1,381 (99.5) |  |  |  |
| At risk | 9,087 | 1,388 |  |  |  |
| **Maternal near-miss at delivery** | | | **193/10,306** | **160/7,962** | **33/2,344** |
| Yes | 164 (1.8) | 29 (2.1) | 0.90  (0.57,1.43)  p = 0.6593 | 0.93  (0.60,1.44)  p = 0.7427 | 0.81  (0.23,2.84)  p = 0.7446 |
| No | 8,807 (98.2) | 1,343 (97.9) |  |  |  |
| At risk | 8,971 | 1,372 |  |  |  |
| **Caesarean delivery** | | | **1,844/10,238** | **1,442/7,962** | **402/2,276** |
| Emergency | 1,589 (17.8) | 265 (19.3) | 1.04  (0.96,1.14)  p = 0.3302 | 1.08  (0.97,1.20)  p = 0.1549 | 0.84 (0.69,1.02)  p = 0.0724 |
| Other delivery modes | 7,317 (82.2) | 1,105 (80.7) |  |  |  |
| At risk | 8,906 | 1,370 |  |  |  |
| **Maternal death** | | | 15/10,428 | 11/8,055 | 4/2,373 |
| Yes | 14 (0.2) | 1 (0.1) | — | — | — |
| No | 9,071 (99.9) | 1,387 (99.9) |  |  |  |
| At risk | 9,085 | 1,388 |  |  |  |
| **Modified maternal morbidity and mortality index (MMMI)** | | | 1,873/10,356 | 1,387/7,996 | 486/2,360 |
| Yes | 1,595 (17.7) | 282 (20.5) | 1.03  (0.88,1.20)  p = 0.6761 | 1.11  (0.92,1.34)  p = 0.2821 | 0.74  (0.56,0.99)  p = 0.0421 |
| No | 7,428 (82.3) | 1,094 (79.5) |  |  |  |
| At risk | 9,023 | 1,376 |  |  |  |
| *^1^* n (%) | | | | | |
| *^2^* Pearson’s Chi-squared test; Fisher’s exact test | | | | | |

^¥^ N appears at multiple rows to reflect change in the denominator due to difference in outcome definitions

§ M/U = Missing or Unknown

£ N (%)

Some outcomes could not be evaluated because of small numbers or lack of model convergence.

**Table S9.** Sensitivity analysis 1 (Possibly infected group added as ‘SARS-CoV-2 infection’): Perinatal and neonatal outcomes by SARS-CoV-2 infection status ^¥^

| **Outcome^§^** | **Confirmed or Probably Infected^£^**  **N = 9,637** | **Confirmed or Probably Uninfected^£^**  **N = 1,455** | **Adjusted random-effects model**  **Relative Risk (95% Confidence Interval); p-value^2^** | | |
| --- | --- | --- | --- | --- | --- |
|  |  |  | **Overall sample**  **(Events / Total)** | **Restricted to pre-Omicron**  **(Events / Total)** | **Restricted to Omicron**  **(Events / Total)** |
| **Stillbirth at/after 22 weeks** | | | **152/10,290** | **105/8,003** | **47/2,287** |
| Yes | 146 (1.6) | 7 (0.5) | 3.58  (0.96,13.44)  p = 0.0585 | 2.68  (0.98,7.34)  p = 0.0545 | — |
| No | 8,810 (98.4) | 1,374 (99.5) |  |  |  |
| At risk | 8,956 | 1,381 |  |  |  |
|  |  |  |  | | |
| **Stillbirth at/after 28 weeks** | | | **140/10,254** | **100/7,983** | **40/2,271** |
| Yes | 134 (1.5) | 7 (0.5) | 3.19  (1.03,9.82)  p = 0.0438 | 2.52  (0.99,6.44)  p = 0.0526 | — |
| No | 8,787 (98.5) | 1,373 (99.5) |  |  |  |
| At risk | 8,921 | 1,380 |  |  |  |
|  |  |  |  | | |
| **Preterm birth (<37 weeks)** | | | **1,503/8,036** | **1,102/6,076** | **401/1,960** |
| Yes | 1,328 (19.9) | 181 (13.2) | 1.48  (1.28,1.71)  p < 0.001 | 1.58  (1.26,1.99)  p = 0.0001 | 1.12  (0.95,1.33)  p = 0.1714 |
| No | 5,356 (80.1) | 1,192 (86.8) |  |  |  |
| At risk | 6,684 | 1,373 |  |  |  |
| **Preterm birth (<34 weeks)** | | | **322/6,953** | **212/5,129** | **110/1,824** |
| Yes | 298 (5.3) | 26 (1.9) | 2.43  (1.66,3.56)  p <0.001 | 2.74  (1.96,3.87)  p <0.001 | 2.17  (1.46,3.23)  p = 0.0001 |
| No | 5,297 (94.7) | 1,347 (98.1) |  |  |  |
| At risk | 5,595 | 1,373 |  |  |  |
| **Preterm birth (<32 weeks)** | | | **136/6,466** | **88/4,728** | **48/1,738** |
| Yes | 129 (2.5) | 9 (0.7) | 2.18  (1.32,3.61)  p = 0.0025 | 4.93  (2.49,9.78)  p <0.001 | 1.38  (1.07,1.78)  p = 0.0142 |
| No | 4,976 (97.5) | 1,364 (99.3) |  |  |  |
| At risk | 5,105 | 1,373 |  |  |  |
| **Low birth weight (<2,500g)** | | | **1,137/9,240** | **871/7,169** | **266/2,072** |
| Yes | 971 (12.2) | 171 (13.0) | 0.996  (0.84,1.19)  p = 0.9640 | 0.993  (0.80,1.24)  p = 0.9484 | 0.91  (0.64,1.31)  p = 0.6077 |
| No | 6,982 (87.8) | 1,141 (87.0) |  |  |  |
| At risk | 7,953 | 1,312 |  |  |  |
| **Very low birth weight (<1,500g)** | | | **216/9,240** | **181/7,168** | **35/2,072** |
| Yes | 180 (2.3) | 38 (2.9) | 1.11  (0.60,2.05)  p = 0.7441 | 1.11  (0.55,2.25)  p = 0.7737 | 1.16 (0.63,2.12)  p = 0.6316 |
| No | 7,773 (97.7) | 1,274 (97.1) |  |  |  |
| At risk | 7,953 | 1,312 |  |  |  |
| **Congenital anomaly (at or after 22 weeks)** | | | **124/6,452** | **107/5,296** | **17/1,156** |
| Yes | 100 (1.8) | 24 (2.3) | 1.07  (0.71,1.62)  p = 0.7421 | 1.20  (0.67,2.15)  p = 0.5451 | 0.39  (0.20,0.75)  p = 0.0050 |
| No | 5,322 (98.2) | 1,032 (97.7) |  |  |  |
| At risk | 5,422 | 1,056 |  |  |  |
| **Congenital anomaly (at or after 28 weeks)** | | | **118/6,426** | **103/5,282** | **15/1,144** |
| Yes | 94 (1.7) | 24 (2.3) | 1.02  (0.69,1.51)  p = 0.9136 | 1.15  (0.66,1.98)  p = 0.6219 | 0.39  (0.18,0.84)  p = 0.0165 |
| No | 5,302 (98.3) | 1,032 (97.7) |  |  |  |
| At risk | 5,396 | 1,056 |  |  |  |
| **Perinatal death (22-weeks stillbirths + early neonatal deaths)** | | | **236/10,290** | **169/8,003** | **67/2,287** |
| Yes | 222 (2.5) | 15 (1.1) | 2,45  (1.22,4.91)  p = 0.0121 | 2.03  (1.30,3.18)  p = 0.0019 |  |
| No | 8,734 (97.5) | 1,366 (98.9) |  |  |  |
| At risk | 8,956 | 6 |  |  |  |
| **Perinatal death (28-weeks stillbirths + early neonatal deaths)** | | | **216/10,254** | **159/7,983** | **57/2,271** |
| Yes | 2023 (2.3) | 14 (1.0) | 2.34  (1.35,4.04)  p = 0.0023 | 2.06  (1.45,2.92)  p = 0.0001 | — |
| No | 8,718 (97.7) | 1,366 (99.0) |  |  |  |
| At risk | 8,921 | 1,380 |  |  |  |
| **Neonatal/infant death** | | | **113/10,059** | **85/7,825** | **28/2,234** |
| Yes | 101 (1.2) | 13 (1.0) | 1.16  (0.55,2.46)  p = 0.7015 | 1.07  (0.52,2.20)  p = 0.8495 | — |
| No | 8,642 (98.8) | 1,346 (99.0) |  |  |  |
| At risk | 8,743 | 1,359 |  |  |  |
| **Neonatal Intensive Care Intensive Unit admission (all infants)** | | | **946/10,040** | **725/7,808** | **221/2,232** |
| Yes | 800 (9.2) | 150 (11.1) | 1.27  (1.09,1.49)  p = 0.0028 | 1.33  (1.12,1.58)  p = 0.0010 | 1.00  (0.65,1.53)  p = 0.9989 |
| No | 7,927 (90.8) | 1,206 (88.9) |  |  |  |
| At risk | 8,727 | 1,356 |  |  |  |
| **Severe Perinatal Morbidity and Mortality Index (SPMMI)** | | | **694/10,170** | **508/7,901** | **186/2,269** |
| Yes | 609 (6.9) | 89 (6.5) | 1.38  (0.993,1.91)  p = 0.0550 | 1.39  (1.01,1.91)  p = 0.0405 | 1.09  (0.63,1.87)  p = 0.7564 |
| No | 8,243 (93.1) | 1,273 (93.5) |  |  |  |
| At risk | 8,852 | 1,362 |  |  |  |
| *^1^* n (%) | | | | | |
| *^2^* Pearson’s Chi-squared test; Fisher’s exact test | | | | | |

^¥^ N appears at multiple rows to reflect change in the denominator due to difference in outcome definitions

§ M/U = Missing or Unknown

£ N (%)

Some outcomes could not be evaluated because of small numbers or lack of model convergence.

**Table S10.** Sensitivity analysis 1 (Possibly infected group added as ‘SARS-CoV-2 infection’): Postpartum outcomes by SARS-CoV-2 infection status

| **Outcome^§^** | **Confirmed or probably infected^£^**  N = 9,637*^1^* | **Confirmed or probably uninfected^£^**  N = 1,455*^1^* | **Adjusted random-effects model**  **Relative Risk (95% Confidence Interval); p-value^2^** | | |
| --- | --- | --- | --- | --- | --- |
|  |  |  | **Overall sample**  **(Events / Total)** | **Restricted to pre-Omicron**  **(Events / Total)** | **Restricted to Omicron**  **(Events / Total)** |
| **Postpartum haemorrhage** | | | **96/9,996** | **84/7,692** | 12/2,304 |
| Yes | 80 (0.9) | 18 (1.4) | 1.11  (0.65,1.89)  p = 0.6998 | 1.10  (0.62,1.97)  p = 0.7479 | — |
| No | 8,651 (99.1) | 1,309 (98.6) |  |  |  |
| At risk | 8,731 | 1,309 |  |  |  |
| **Postpartum complications: Maternal infection or endometritis** | | | **183/9,872** | **143/7,642** | **40/2,230** |
| Yes | 161 (1.9) | 25 (1.9) | 1.22  (0.91,1.62)  p = 0.1807 | 1.35  (0.90,2.03)  p = 0.1513 | 0.94  (0.60,1.47)  p = 0.7748 |
| No | 8,450 (98.1) | 1,278 (98.1) |  |  |  |
| At risk | 8,611 | 1,303 |  |  |  |
| **Any readmission in the postpartum period** | | | **106/9,891** | **82/7,658** | **25/2,233** |
| Yes | 87 (1.0) | 21 (1.6) | 0.93  (0.67,1.28)  p = 0.6469 | 0.81  (0.57,1.15)  p = 0.2437 | 1.60  (0.45,5.68)  p = 0.4664 |
| No | 8,542 (99.0) | 1,283 (98.4) |  |  |  |
| At risk | 8,629 | 1,304 |  |  |  |
| *^1^* n (%) | | | | | |
| *^2^* Pearson’s Chi-squared test | | | | | |

§ M/U = Missing or Unknown

£ N (%)

Some outcomes could not be evaluated because of small numbers or lack of model convergence.

**Table S11.** Sensitivity analysis 2 (Possibly infected group added as ‘non-infection’): Maternal and pregnancy outcomes by SARS-CoV-2 infection status^¥^

| **Outcome^§^** | **Confirmed or Probably Infected^£^**  N = 2,303*^1^* | **Confirmed or Probably Uninfected^£^**  N = 8,787*^1^* | **Adjusted (Infected vs Uninfected) random-effects model**  **Adjusted Relative Risk (95% Confidence Interval); p-value^2^** | | |
| --- | --- | --- | --- | --- | --- |
|  |  |  | **Overall sample**  **(Events / Total)** | **Restricted to pre-Omicron**  **Period**  **(Events / Total)** | **Restricted to Omicron**  **Period**  **(Events / Total)** |
| **Miscarriage (before 22 weeks)** | | | **85/2,2027** | **36/1,095** | **49/932** |
| Yes | 11 (3.7) | 74 (4.3) | 0.67  (0.49,0.91)  p = 0.0110 | 0.79  (0.38,1.67)  p = 0.5430 | 0.73  (0.55,0.97)  p = 0.0282 |
| No | 290 (96.4) | 1,656 (95.7) |  |  |  |
| At risk | 301 | 1,730 |  |  |  |
| **Miscarriage (before 28 weeks)** | | | **92/2,036** | **38/1,098** | **54/938** |
| Yes | 11 (3.6) | 82 (4.7) | 0.65  (0.47,0.90)  p = 0.0088 | 0.73  (0.36,1.48)  p = 0.3899 | 0.72  (0.54,0.95)  p = 0.0220 |
| No | 292 (96.4) | 1,656 (95.3) |  |  |  |
| At risk | 303 | 1,738 |  |  |  |
| **Haemorrhage (Antepartum, Intrapartum, or Postpartum)** | | | **248/10,300** | **176/8,007** | **72/2,293** |
| Yes | 80 (3.6) | 171 (2.1) | 2.10  (1.41,3.15)  p = 0.0003 | 2.46  (1.90,3.18)  p < 0.001 | 1.09  (0.41,2.90)  p = 0.8561 |
| No | 2,126 (96.5) | 7,931 (97.9) |  |  |  |
| At risk | 2,206 | 8,102 |  |  |  |
| **Preeclampsia/eclampsia** | | | **544/10,436** | **415/8,063** | **129/2,373** |
| Yes | 156 (7.0) | 389 (4.7) | 1.10  (0.72,1.70)  p = 0.6532 | 1.11  (0.70,1.76)  p = 0.6551 | 1.10  (0.73,1.66)  0.6471 |
| No | 2,085 (93.0) | 7,851 (95.3) |  |  |  |
| At risk | 2,241 | 8,240 |  |  |  |
| **Hypertensive disorders of pregnancy** | | | **711/10,438** | **533/8,065** | **178/2,373** |
| Yes | 218 (9.7) | 495 (6.0) | 1.22  (0.85,1.75)  p = 0.2801 | 1.24  (0.91,1.70)  p = 0.1816 | 1.23  (0.75,2.02)  p = 0.4080 |
| No | 2,024 (90.3) | 7,746 (94.0) |  |  |  |
| At risk | 2,242 | 8,241 |  |  |  |
| **Thromboembolic disease** | | | **12/10,437** | **11/8,064** | **1/2,373** |
| Yes | 4 (0.2) | 8 (0.1) | 1.63  (0.54,4.90)  p = 0.3833 | 1.81  (0.59,5.57)  p = 0.3029 | — |
| No | 2,236 (99.8) | 8,231 (99.9) |  |  |  |
| At risk | 2,240 | 8,239 |  |  |  |
| **Preterm labour** | | | **589/7,734** | **409/5,679** | **180/2,055** |
| Yes | 118 (7.5) | 472 (7.6) | 0.93  (0.59,1.47)  p = 0.7693 | 0.89  (0.54,1.46)  p = 0.6446 | 1.13  (0.68,1.88)  p = 0.6343 |
| No | 1,459 (92.5) | 5,076 (92.4) |  |  |  |
| At risk | 1,577 | 6,178 |  |  |  |
| **Placental abruption** | | | 47/10,433 | 30/8,061 | 17/2,372 |
| Yes | 10 (0.5) | 37 (0.5) | 1.19  (0.52,2.68)  p = 0.6838 | 1.31  (0.63,2.71)  p = 0.4687 | 0.80  (0.12,5.35)  p = 0.8141 |
| No | 2,230 (99.6) | 8,198 (99.5) |  |  |  |
| At risk | 2,240 | 8,235 |  |  |  |
| **Maternal near-miss at delivery** | | | **193/10,306** | **160/7,962** | **33/2,344** |
| Yes | 36 (1.7) | 157 (1.9) | 1.40  (0.98,1.99)  p = 0.0646 | 1.52  (1.05,2.19)  p = 0.0267 | 0.71  (0.35,1.44)  0.3376 |
| No | 2,143 (98.4) | 8,007 (98.1) |  |  |  |
| At risk | 2,179 | 8,164 |  |  |  |
| **Caesarean delivery** | | | **1,844/10,238** | **1,442/7,962** | **402/2,276** |
| Emergency | 506 (23.0) | 1,348 (16.7) | 1.19  (0.91,1.56)  p = 0.2038 | 1.24  (0.98,1.58)  p = 0.0722 | 0.94  (0.73,1.22)  p = 0.6551 |
| Other delivery modes | 1,694 (77.0) | 6,728 (83.3) |  |  |  |
| At risk | 2,200 | 8,076 |  |  |  |
| **Maternal death** | | |  |  |  |
| Yes | 8 (0.4) | 7 (0.1) | — | — | — |
| No | 2,230 (99.6) | 8,228 (99.9) |  |  |  |
| At risk | 2,238 | 8,235 |  |  |  |
| **Modified maternal morbidity and mortality index (MMMI)** | | | 1,873/10,356 | 1,387/7,996 | 486/2,360 |
| Yes | 465 (21.1) | 1,412 (17.2) | 1.16  (0.97,1.39)  p = 0.1146 | 1.23  (1.09,1.37)  p = 0.0004 | 1.00 (0.64,1.56)  p = 0.9850 |
| No | 1,741 (78.9) | 6,781 (82.8) |  |  |  |
| At risk | 2,206 | 8,193 |  |  |  |
| *^1^* n (%) | | | | | |
| *^2^* Pearson’s Chi-squared test; Fisher’s exact test | | | | | |

^¥^ N appears at multiple rows to reflect change in the denominator due to difference in outcome definitions

§ M/U = Missing or Unknown

£ N (%)

Some outcomes could not be evaluated because of small numbers or lack of model convergence.

**Table S12.** Sensitivity analysis 2 (Possibly infected group added as ‘non-infection’): Perinatal and neonatal outcomes by SARS-CoV-2 infection status ^¥^

| **Outcome^§^** | **Confirmed or Probably Infected^£^**  **N = 2,303** | **Confirmed or Probably Uninfected^£^**  **N = 8,787** | **Adjusted random-effects model**  **Adjusted Relative Risk (95% Confidence Interval); p-value^2^** | | |
| --- | --- | --- | --- | --- | --- |
|  |  |  | **Overall sample**  **(Events / Total)** | **Restricted to pre-Omicron**  **(Events / Total)** | **Restricted to Omicron**  **(Events / Total)** |
| **Stillbirth at/after 22 weeks )** | | | **152/10,290** | **105/8,003** | **47/2,287** |
| Yes | 15 (0.7) | 138 (1.7) | 0.53  (0.33,0.86)  p = 0.0094 | 0.59  (0.42,0.84)  p = 0.0030 | 0.25  (0.04,1.53)  p = 0.1343 |
| No | 2,203 (99.3) | 7,979 (98.3) |  |  |  |
| At risk | 2,218 | 8,117 |  |  |  |
| **Stillbirth at/after 28 weeks** | | | **140/10,254** | **100/7,983** | **40/2,271** |
| Yes | 14 (0.6) | 127 (1.6) | 0.52  (0.32,0.87)  p = 0.0128 | 0.55  (0.36,0.82)  p = 0.0038 | 0.30  (0.07,1.37)  p = 0.1196 |
| No | 2,190 (99.4) | 7,968 (98.4) |  |  |  |
| At risk | 2,204 | 8,095 |  |  |  |
| **Preterm birth (<37 weeks)** | | | **1,503/8,036** | **1,102/6,076** | **401/1,960** |
| Yes | 361 (23.4) | 1,147 (17.6) | 1.15  (0.92,1.44)  p = 0.2142 | 1.26  (0.96,1.65)  p = 0.0989 | 0.95 (0.69,1.30)  p = 0.7428 |
| No | 1,181 (76.6) | 5,367 (82.4) |  |  |  |
| At risk | 1,542 | 6,514 |  |  |  |
| **Preterm birth (<34 weeks** | | | **322/6,953** | **212/5,129** | **110/1,824** |
| Yes | 95 (7.4) | 229 (4.0) | 1.46 (0.88,2.43)  p = 0.1435 | 1.90  (1.02,3.56)  p = 0.0446 | 1.02  (0.62,1.66)  p = 0.9625 |
| No | 1,195 (92.6) | 5,448 (96.0) |  |  |  |
| At risk | 1,290 | 5,677 |  |  |  |
| **Preterm birth (<32 weeks)** | | | **136/6,466** | **88/4,728** | **48/1,738** |
| Yes | 50 (4.3) | 88 (1.7) | 1.67  (1.02,2.73)  p = 0.0415 | 3.35  (1.68,6.69)  p = 0.0006 | 0.85  (0.70,1.05)  p = 0.1251 |
| No | 1,123 (95.7) | 5,216 (98.3) |  |  |  |
| At risk | 1,173 | 5,304 |  |  |  |
| **Low birth weight (<2,500g)** | | | **1,137/9,240** | **871/7,168** | **266/2,072** |
| Yes | 286 (15.1) | 855 (11.6) | 1.27  (1.09,1.48)  p = 0.0022 | 1.33  (1.10,1.62)  p = 0.0037 | 1.09  (0.88,1.35)  p = 0.4487 |
| No | 1,607 (84.9) | 6,515 (88.4) |  |  |  |
| At risk | 1,893 | 7,370 |  |  |  |
| **Very low birth weight (<1,500g)** | | | **216/9,240** | **181/7,168** | **35/2,072** |
| Yes | 51 (2.7) | 167 (2.3) | 1.16  (0.68,1.98)  p = 0.5796 | 1.28  (0.67,2.44)  p = 0.4543 | 0.92 (0.47, 1.79) |
| No | 1,842 (97.3) | 7,203 (97.7) |  |  |  |
| At risk | 1,893 | 7,370 |  |  |  |
| **Congenital anomaly (at or after 22 weeks)** | | | **124/6,452** | **107/5,296** | **17/1,156** |
| Yes | 63 (3.7) | 61 (1.3) | 2.81  (1.68,4.69)  p = 0.0001 | 2.87  (1.51,5.46)  p = 0.0014 | 2.15  (1.16,3.98)  p = 0.0145 |
| No | 1,645 (96.3) | 4,707 (98.7) |  |  |  |
| At risk | 1,708 | 4,768 |  |  |  |
| **Congenital anomaly (at or after 28 weeks)** | | | **118/6,426** | **103/5,282** | **15/1,144** |
| Yes | 59 (3.7) | 59 (1.2) | 2.62  (1.64,4.19)  p = 0.0001 | 2.69  (1.49,4.84)  p = 0.0010 | 1.95  (1.02,3.72)  p = 0.0432 |
| No | 1,637 (96.5) | 4,695 (98.8) |  |  |  |
| At risk | 1,696 | 4,754 |  |  |  |
| **Perinatal death (22-weeks stillbirths + early neonatal deaths)** | | | **236/10,290** | **169/8,003** | **67/2,287** |
| Yes | 27 (1.2) | 210 (2.6) | 0.64  (0.38,1.09)  p = 0.0989 | 0.82  (0.56,1.22)  p = 0.3360 | 0.14  (0.02,1.04)  p = 0.0543 |
| No | 2,191 (98.8) | 7,907 (97.4) |  |  |  |
| At risk | 2,218 | 8,117 |  |  |  |
| **Perinatal death (28-weeks stillbirths + early neonatal deaths)** | | | **216/10,254** | **159/7,983** | **57/2,271** |
| Yes | 24 (1.1) | 193 (2.4) | 0.62  (0.33,1.15)  p = 0.1298 | 0.75  (0.46,1.24)  p = 0.2594 | 0.17  (0.03,1.02)  p = 0.0530 |
| No | 2,180 (98.9) | 7,902 (97.6) |  |  |  |
| At risk | 2,204 | 8,095 |  |  |  |
| **Neonatal/infant death** | | | **113/10,059** | **85/7,825** | **28/2,234** |
| Yes | 20 (0.9) | 94 (1.2) | 0.82  (0.29,2.29)  p = 0.7036 | 1.20  (0.55,2.63)  p = 0.6403 | — |
| No | 2,152 (99.1) | 7,834 (98.8) |  |  |  |
| At risk | 2,172 | 7,928 |  |  |  |
| **Neonatal Intensive Care Unit admission (all infants)** | | | **946/10,040** | **725/7,808** | **221/2,232** |
| Yes | 321 (14.9) | 629 (7.9) | 1.45  (0.99,2.14)  p = 0.0581 | 1.71  (1.19,2.46)  p = 0.0035 | 0.83  (0.70,0.99)  p = 0.0342 |
| No | 1,840 (85.2) | 7,291 (92.1) |  |  |  |
| At risk | 2,161 | 7,920 |  |  |  |
| **Severe Perinatal Morbidity and Mortality Index** | | | **694/10,170** | **508/7,901** | **186/2,269** |
| Yes | 196 (9.1) | 502 (6.2) | 1.28  (0.94,1.76)  p = 0.1178 | 1.50  (1.07,2.10)  p = 0.0179 | 0.71  (0.43,1.17)  p = 0.1826 |
| No | 1,967 (90.9) | 7,547 (93.8) |  |  |  |
| At risk | 2,163 | 8,049 |  |  |  |
| *^1^* n (%) | | | | | |
| *^2^* Pearson’s Chi-squared test; Fisher’s exact test | | | | | |

^¥^ N appears at multiple rows to reflect change in the denominator due to difference in outcome definitions

§ M/U = Missing or Unknown

£ N (%)

Some outcomes could not be evaluated because of small numbers or lack of model convergence.

**Table S13.** Sensitivity analysis 2 (Possibly infected group added as ‘non-infection’): Postpartum outcomes by SARS-CoV-2 infection status

| **Outcome^§^** | **Confirmed or Probably Infected^£^**  N = 2,305*^1^* | **Confirmed or Probably Uninfected^£^**  N = 8,787*^1^* | **Adjusted random-effects model**  **Adjusted Relative Risk (95% Confidence Interval); p-value^2^** | | |
| --- | --- | --- | --- | --- | --- |
|  |  |  | **Overall sample**  **(Yes / No)** | **Restricted to pre-Omicron**  **(Yes / No)** | **Restricted to Omicron**  **(Yes / No)** |
| **Postpartum haemorrhage** | | | **96/9,996** | **84/7,692** | **12/2,304** |
| Yes | 12 (0.6) | 86 (1.1) | 0.63  (0.44,0.90)  p = 0.0105 | 0.54  (0.35,0.82)  p = 0.0041 | 0.97  (0.50,1.87)  p = 0.9158 |
| No | 2,060 (99.4) | 7,880 (98.9) |  |  |  |
| At risk | 2,072 | 7,966 |  |  |  |
| **Postpartum complications: Maternal infection or endometritis** | | | **183/9,872** | **143/7,642** | **40/2,230** |
| Yes | 53 (2.6) | 133 (1.7) | 1.28  (0.95,1.73)  p = 0.1064 | 1.19  (0.78,1.81)  p = 0.4112 | 1.61  (1.08,2.41)  p = 0.0198 |
| No | 1,998 (97.4) | 7,728 (97.4) |  |  |  |
| At risk | 2,051 | 7,861 |  |  |  |
| **Any readmission in the postpartum period** | | | **106/9,891** | **82/7,658** | **25/2,233** |
| Yes | 34 (1.7) | 73 (0.9) | 1.33  (0.74,2.41)  p = 0.3438 | 1.32  (0.57,3.06)  p = 0.5238 | 1.56  (0.70,3.46)  p = 0.2738 |
| No | 2,032 (98.4) | 7,865 (99.1) |  |  |  |
| At risk | 2,066 | 7,865 |  |  |  |
| *^1^* n (%) | | | | | |

§ M/U = Missing or Unknown

£ N (%)

Some outcomes could not be evaluated because of small numbers or lack of model convergence.

**Supplementary Material 17**

**Fig S4**. Proportion of symptoms by COVID-19 variant of concern period, among those with SARS-CoV-2 infection during pregnancy

**Table S14**. Maternal, perinatal and postpartum outcomes by illness severity, among those with SARS-CoV-2 infection during pregnancy

| **Outcome** | **Adjusted random-effects model^£^** | | |
| --- | --- | --- | --- |
|  | **Asymptomatic or Mild infection**  **N = 1,891** | **Moderate or Severe infection**  **N = 313** | **Adjusted Relative Risk (95% Confidence Interval); p-value** |
|  | **N (%)** | **N (%)** |  |
| **Haemorrhage (antepartum, intrapartum, or immediately postpartum)** | | | |
| Yes | 65 (3.5) | 14 (4.6) | 1.21 (0.71, 2.06)  p = 0.4878 |
| No | 1,805 (96.5) | 293 (95.4) |  |
| At risk | 1,870 | 307 |  |
| **Hypertensive disorders of pregnancy** | | | |
| Yes | 182 (9.6) | 33 (10.6) | 0.87 (0.58, 1.30)  p = 0.4859 |
| No | 1,706 (90.4) | 279 (89.4) |  |
| At risk | 1,888 | 312 |  |
| **Maternal near-miss at delivery** | | | |
| Yes | 22 (1.2) | 13 (4.4) | 3.42 (2.18,5.36)  p < 0.001 |
| No | 1,818 (98.8) | 284 (95.6) |  |
| At risk | 1,840 | 297 |  |
| **Caesarean delivery** | | | |
| Emergency | 397 (21.4) | 104 (34.1) | 1.33 (1.05, 1.69)  p = 0.0169 |
| Other delivery modes^α^ | 1,456 (78.6) | 201 (65.9) |  |
| At risk | 1,853 | 305 |  |
| **Modified maternal morbidity and mortality index (MMMI)** | | | |
| Yes | 368 (19.7) | 89 (29.6) | 1.24 (0.99, 1.56)  p = 0.0593 |
| No | 1,495 (80.3) | 212 (70.43) |  |
| At risk | 1,863 | 301 |  |
| **Preterm birth (<37 weeks)** | | | |
| Yes | 275 (21.7) | 81 (31.4) | 1.38 (0.85, 2.22)  p = 0.1891 |
| No | 994 (78.3) | 177 (6863) |  |
| At risk | 1,269 | 258 |  |
| **Preterm birth (<34 weeks)** | | | |
| Yes | 74 (7.0) | 21 (9.3) | 1.23 (0.59, 2.55)  p = 0.5835 |
| No | 982 (93.0) | 204 (90.7) |  |
| At risk | 1,056 | 225 |  |
| **Preterm birth (<32 weeks)** | | | |
| Yes | 38 (4.0) | 12 (5.9) | 1.37 (0.69, 2.73)  p = 0.3668 |
| No | 923 (96.0) | 192 (94.1) |  |
| At risk | 961 | 204 |  |
| **Congenital anomaly (at or after 22 weeks)** | | | |
| Yes | 55 (3.8) | 7 (2.9) | 0.53 (0.17,1.62)  p = 0.2623 |
| No | 1,392 (96.2) | 230 (97.1) |  |
| At risk | 1,447 | 237 |  |
| **Congenital anomaly (at or after 28 weeks)** | | | |
| Yes | 53 (3.7) | 5 (2.2) | 0.36 (0.11,1.25)  p = 0.1085 |
| No | 1,387 (96.3) | 227 (97.8) |  |
| At risk | 1,440 | 232 |  |
| **NICU admission** | | | |
| Yes | 255 (14.0) | 61 (20.6) | 1.12 (0.58, 2.18)  p = 0.7406 |
| No | 1,568 (86.0) | 235 (79.4) |  |
| At risk | 1,823 | 296 |  |
| **Severe Perinatal Morbidity and Mortality Index (SPMMI)** | | | |
| Yes | 155 (8.5) | 39 (13.2) | 1.17 (0.52, 2.63)  p = 0.7041 |
| No | 1,673 (91.5) | 256 (86.8) |  |
| At risk | 1,828 | 295 |  |

^¥^ For each outcome, models were adjusted for maternal age at enrollment (years) and presence of medical conditions (none vs. one or more). Only the relative risks for illness severity are presented in the table.

**Country-specific ethical approvals**

**Argentina:** Ethical approval obtained from the Pan American Health Organization Ethics Review Committee (PAHOERC) on 4 November 2021 (Ref: PAHOERC.0428.03), and from the following local ethics committees: Comité de Ética Independiente del CREP on 22 September 2021; Ref 3_21, Comité de Ética del Hospital Materno Neonatal “Eloisa Torrent de Vidal” on 14 June 2021, and Comité de Ética del Hospital Privado – Centro Médico de Córdoba on 10 September 2021; Ref HP4-346.

**Brazil:** Ethical approval obtained from PAHOERC on 2 July 2021 (Ref: PAHOERC.0369.02) and on 23 March 2023, and from the following local ethics committee for all centers: Comissão Nacional de Ética em Pesquisa (CONEP) on 30 June 2021 (Ref: 4.817.831), with amendments approved on 6 June 2023.

**Burkina Faso:** Ethical approval requirements were waived by the WHO African Region Ethics Review Committee (AFRO ERC) on 3 August 2021 (Ref: AFR/ERC/2021/8.2) and on 23 June 2023 (Ref: AFR/ERC/2023/6.10). Local approval was obtained from the Comité d’éthique pour la recherche en Santé (CERS) on 2 June 2021, with amendments approved on 7 June 2023.

**Chile:**  Ethical approval was obtained from the World Health Organization (WHO) / Pan American Health Organization (PAHO) Ethics Review Committee (Ref. PAHOERC.0590.02; approval dated 16 December 2022), as well as from the following local ethics committees in Chile: Comité de Ética Científica de la Dirección de Servicio de Salud Araucanía Sur (approval dated 21 October 2021; Ref. 244), Comité de Ética Científica de la Dirección de Servicio de Salud Araucanía Sur (approval dated 23 March 2023; Ref. 57), Comité Ético Científico del Servicio de Salud del Reloncaví – Hospital Puerto Montt (approval dated 26 July 2021; Ref. 16), and Dirección de Servicio de Salud Araucanía Sur – Hospital Dr. Hernán Henríquez Aravena (approval dated 30 March 2023; Ref. 2553).

**Ghana:** WHO AFRO ERC approval obtained on 26 February 2021 (Ref: AFR/ERC/2021/2.2), with local approvals granted by the Ghana Health Service Ethics Review Committee on 26 April 2021 and 11 November 2021 (Ref: GHSERC 017/02/21), and by the University of Ghana IRB on 28 October 2021 (IRB00006220).

**Kenya:** WHO AFRO ERC approval on 25 February 2021 (Ref: AFR/ERC/2021/2.4) with continuing approval on 14 April 2023 (Ref: AFR/ERC/2023/4.1). Local approvals from Aga Khan University, Nairobi on 12 May 2021 (Ref: 2020/IERC-153 (v2)) and 6 March 2023 (Ref: 2020/IERC-153 (V3)), and from the National Commission for Science, Technology and Innovation (NACOSTI) on 9 June 2021 (Ref: 336896).

**Pakistan:** WHO Eastern Mediterranean Regional Ethics Review Committee (EMRERC) approval with waiver in 2021 and 10 December 2023. Local approvals obtained from the Aga Khan University Ethics Review Committee on 26 February 2023 (Ref: 2023-5838-24122), with amendments approved on 17 February 2022 (Ref: 2022-5838-20665), 18 May 2022 (Ref: 2022-5838-21504), and 8 November 2022 (Ref: 2022-5838-23158). National Bioethics Committee approvals were granted on 23 February 2021 (Ref: 4-87/COVID-62/NBC/21), with amendment and extension approvals on 26 May 2021 and 5 September 2022 (Ref: 4-87/COVID-62/22/294).

**Philippines:** WHO Western Pacific Regional Ethics Review Committee (WPRO-ERC) approval on 27 August 2021 (Ref: 2021.14.PHL.3.MCN). Local approvals from the Single Joint Research Ethics Board (SJREB) on 19 May 2021 (Ref: SJREB-2020-30) and 20 February 2023 (amendment), the University of the Philippines Manila Research Ethics Board (UPMREB) on 9 June 2021 (Ref: 2020-320-01-SJREB) and 10 March 2023 (continuing review; Ref: IRB 2020-18-4), and the Research Institute for Tropical Medicine – Department of Health on 11 June 2021 (Ref: IRB 2020-18-2). 
**Spain:** Comité Ético de Investigación de la Dirección General de Salud Pública y Centro Superior de Investigación en Salud Pública approval obtained on 21 December 2020 (Ref: 20201221/06), and Comité Ético de Investigación Clínica del Hospital Universitario Doctor Peset de Valencia approval obtained on 29 November 2021.

**Tunisia:** EMRERC approvals (with waiver) on 19 October 2021 and 3 March 2023, on the basis of local approvals obtained from: the Comité d’éthique du Centre de Maternité et de Néonatologie de Tunis on 23 July 2021, and from the Comité d’éthique Biomédicale de l’Institut Pasteur de Tunis on 29 July 2021 (Ref: 2021/12/I/LR16IPT/V1), with amendment approval on 26 June 2023 (Ref: 2022/12/I/LR16IPT/Amendment/V1).

**Informed Consent:** Obtained from all study participants prior to enrolment in the study, and in accordance with WHO ERCs and local ethics committee requirements.
